# Changing pattern of the genetic diversities of *Plasmodium falciparum* merozoite surface protein-1 and merozoite surface protein-2 in Myanmar isolates

**DOI:** 10.1186/s12936-019-2879-7

**Published:** 2019-07-16

**Authors:** Hương Giang Lê, Jung-Mi Kang, Hojong Jun, Jinyoung Lee, Thị Lam Thái, Moe Kyaw Myint, Khin Saw Aye, Woon-Mok Sohn, Ho-Joon Shin, Tong-Soo Kim, Byoung-Kuk Na

**Affiliations:** 1 Department of Parasitology and Tropical Medicine, and Institute of Health Sciences, Gyeongsang National University College of Medicine, Jinju, 52727 Republic of Korea; 2 BK21Plus Team for Anti-aging Biotechnology and Industry, Department of Convergence Medical Science, Gyeongsang National University, Jinju, 52727 Republic of Korea; 3 Department of Tropical Medicine, and Inha Research Institute for Medical Sciences, Inha University College of Medicine, Incheon, 22212 Republic of Korea; 4 Department of Medical Research Pyin Oo Lwin Branch, Pyin Oo Lwin, Myanmar; 5 Department of Microbiology, Ajou University School of Medicine, Suwon, 16499 Republic of Korea

**Keywords:** *Plasmodium falciparum*, Merozoite surface protein-1, Merozoite surface protein-2, Genetic diversity, Myanmar

## Abstract

**Background:**

*Plasmodium falciparum* merozoite surface protein-1 (PfMSP-1) and -2 (PfMSP-2) are major blood-stage vaccine candidate antigens. Understanding the genetic diversity of the genes, *pfmsp*-*1* and *pfmsp*-*2*, is important for recognizing the genetic structure of *P. falciparum*, and the development of an effective vaccine based on the antigens. In this study, the genetic diversities of *pfmsp*-*1* and *pfmsp*-*2* in the Myanmar *P. falciparum* were analysed.

**Methods:**

The *pfmsp*-*1* block 2 and *pfmsp*-*2* block 3 regions were amplified by polymerase chain reaction from blood samples collected from Myanmar patients who were infected with *P. falciparum* in 2013–2015. The amplified gene fragments were cloned into a T&A vector, and sequenced. Sequence analysis of Myanmar *pfmsp*-*1* block 2 and *pfmsp*-*2* block 3 was performed to identify the genetic diversity of the regions. The temporal genetic changes of both *pfmsp*-*1* and *pfmsp*-*2* in the Myanmar *P. falciparum* population, as well as the polymorphic diversity in the publicly available global *pfmsp*-*1* and *pfmsp*-*2*, were also comparatively analysed.

**Results:**

High levels of genetic diversity of *pfmsp*-*1* and *pfmsp*-*2* were observed in the Myanmar *P. falciparum* isolates. Twenty-eight different alleles of *pfmsp*-*1* (8 for K1 type, 14 for MAD20 type, and 6 for RO33 type) and 59 distinct alleles of *pfmsp*-*2* (18 for FC27, and 41 for 3D7 type) were identified in the Myanmar *P. falciparum* population in amino acid level. Comparative analyses of the genetic diversity of the Myanmar *pfmsp*-*1* and *pfmsp*-*2* alleles in the recent (2013–2015) and past (2004–2006) Myanmar *P. falciparum* populations indicated the dynamic genetic expansion of the *pfmsp*-*1* and *pfmsp*-*2* in recent years, suggesting that a high level of genetic differentiation and recombination of the two genes may be maintained. Population genetic structure analysis of the global *pfmsp*-*1* and *pfmsp*-*2* also suggested that a high level of genetic diversity of the two genes was found in the global *P. falciparum* population.

**Conclusion:**

Despite the recent remarkable decline of malaria cases, the Myanmar *P. falciparum* population still remains of sufficient size to allow the generation and maintenance of genetic diversity. The high level of genetic diversity of *pfmsp*-*1* and *pfmsp*-*2* in the global *P. falciparum* population emphasizes the necessity for continuous monitoring of the genetic diversity of the genes for better understanding of the genetic make-up and evolutionary aspect of the genes in the global *P. falciparum* population.

**Electronic supplementary material:**

The online version of this article (10.1186/s12936-019-2879-7) contains supplementary material, which is available to authorized users.

## Background

Although global malaria cases have remarkably decreased in recent years, malaria is still one of the most important public health concerns worldwide, with an estimated 219 million cases and 435,000 related deaths in 2016 [1]. Many efforts have been undertaken to develop an effective malaria vaccine, but to date, there is no available licensed malaria vaccine. The Greater Mekong Subregion (GMS) has long been one of the most malarious regions in the world [2]. Among the countries in GMS, Myanmar has the highest malaria burden, accounting for an estimated 77% of malaria cases, and approximately 79% of malaria deaths in the GMS [3]. Several interventions have been made in the country to reduce the malaria burden, including the training and deployment of community health workers, the distribution of insecticide-treated bed nets, strategies to improve access to rapid diagnostic tests, and the provision of artemisinin-based combination therapy (ACT) [4]. Due to these nationwide efforts, the annual incidence of malaria in Myanmar has been greatly reduced, with an 81.1% decline between the years 2005 and 2014. However, the recent rise and spread of artemisinin resistance parasite is the greatest threat to the effective control and elimination of malaria in the country [5].

Understanding the population genetic structure of malaria parasites is necessary to determine the epidemiology, diversity, distribution, and dynamics of the natural population of malaria parasites. *Plasmodium falciparum* merozoite surface protein-1 (PfMSP-1) and -2 (PfMSP-2) are major blood-stage vaccine candidate antigens, which play important roles in erythrocyte invasion [6, 7], and are targeted by host immune responses [8–12]. They show high polymorphic patterns in different geographical settings, and have been considered as suitable polymorphic markers for genotyping genetically distinct *P. falciparum* sub-populations [13–17]. PfMSP-1 is initially synthesized as a large molecular size precursor with an approximate size of 190 kDa, and then undergoes post-translational proteolytic processing into four fragments, 83 kDa, 30 kDa, 38 kDa and 42 kDa [18]. These fragments persist as a non-covalent linked complex on the surface of mature merozoites [19, 20]. The *pfmsp*-*1* is divided into 17 distinct blocks that are conserved, semi-conserved, and variable [19, 21, 22], among which block 2 is the most polymorphic part of the gene, and is grouped into three allelic types, namely K1, MAD20, and RO33, based on their sequence polymorphic patterns [21, 23]. PfMSP-2 is a glycoprotein that consists of highly polymorphic central repeats (block 3), flanked by unique variable domains (block 2 and block 4), and conserved N- and C-terminal regions. The *pfmsp*-*2* alleles are in general grouped into two dimorphic families, FC27 and 3D7, which are based on block 2 and block 4, while repeat regions in block 3 also differ in the number and sequence of repeat units [24–26].

Similar to the *P. falciparum* population in other malaria endemic areas, the Myanmar *P. falciparum* population has also shown high levels of genetic diversity [26–29]. Extensive genetic diversity with diverse allele types was also previously identified in *pfmsp*-*1* and *pfmsp*-*2* in *P. falciparum* Myanmar isolates that were collected in 2004–2006 [26]. This study analysed the genetic polymorphisms of *pfmsp*-*1* block 2 and *pfmsp*-*2* block 3 in Myanmar *P. falciparum* isolates that were collected in 2013–2015. They were also compared with the sequences from previous years 2004–2006, to understand the temporal changes of genetic heterogeneity of the two genes in the Myanmar *P. falciparum* population. Comparative analysis of the global *pfmsp*-*1* and *pfmsp*-*2* was also performed, in order to gain in-depth understanding of the genetic make-up of the two genes in the global *P. falciparum* population.

## Methods

### Blood samples and genomic DNA extraction

**Fig. 1:**
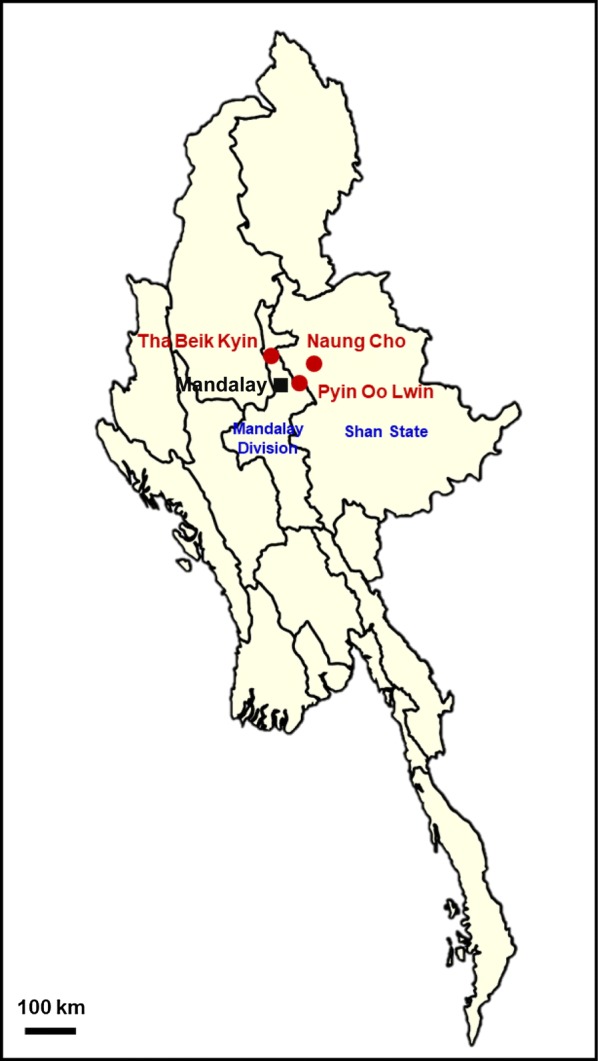
Map of study sites. Community-based survey was performed in three villages in Naung Cho, Tha Beik Kyin and Pyin Oo Lwin, Myanmar from 2013 to 2015. Blood samples were collected from patients who infected *P. falciparum* and used in this study

A total of 115 blood samples used in this study were obtained from *P. falciparum* infected symptomatic patients who live in villages located in Naung Cho, Tha Beik Kyin, and Pyin Oo Lwin, Myanmar during community-based surveys from 2013 to 2015 (Fig. 1). Malaria transmission in these areas is heterogeneous and seasonal, and most malaria cases occur at the peak during and just after the rainy season. *P. falciparum* infection was confirmed by Giemsa-stained thick and thin blood smear examination. All *P. falciparum* positive samples were further confirmed by polymerase chain reaction (PCR) targeting 18S ribosomal RNA (rRNA) gene [30]. Prior to drug treatment, patient’s blood samples were collected on filter papers (Whatman 3 mm, GE Healthcare, Pittsburg, USA), air-dried, and stored in sealed plastic bags at ambient temperature, until use. Informed consent was obtained from all the patients before blood collection. The study protocol was approved by either the Ethics committee of the Ministry of Health, Myanmar (97/Ethics 2015), or the Biomedical Research Ethics Review Board of Inha University School of Medicine, Republic of Korea (INHA 15-013).

### Amplification and sequence analysis of *pfmsp*-*1* and *pfmsp*-*2*

Genomic DNA was purified from the blood filters by using a QIAamp DNA Blood Kit (Qiagen, Hilden, Germany), following the manufacturer’s instructions. The primers specific for the block 2 of *pfmsp*-*1* and block 3 of *pfmsp*-*2* were designed as described previously [26, 31]. The two genes were amplified by a nested PCR method. Each amplification was done with thermal cycling conditions of 94 °C for 5 min; and 30 cycles of 94 °C for 1 min, 52 °C for 1 min, and 72 °C for 1 min 30 s, followed by a final extension at 72 °C for 10 min. Ex *Taq* DNA polymerase (Takara, Otsu, Japan) with proof-reading activity was used in all PCR amplification steps to minimize the nucleotide mis-incorporation. Each PCR product was resolved on a 1.5% agarose gel, and was visualized under ultraviolet (UV). The multiplicity of infection (MOI) was estimated by the average number of PCR fragments for the corresponding locus per infected individual [26]. The PCR products were purified from the gel, and cloned into the T&A cloning vector (Real Biotech Corporation, Banqiao City, Taiwan). Each ligation mixture was transformed into *Escherichia coli* DH5α competent cells, and colonies were screened for the presence of appropriate insert by colony PCR. The nucleotide sequences of the positive clones of *pfmsp*-*1* and *pfmsp*-*2* were analysed through automatic DNA sequencing with M13 forward and M13 reverse primers by the Sanger methods. Plasmids from at least two independent clones from each transformation mixture were sequenced in both directions, in order to verify the sequence accuracy. The nucleotide and deduced amino acid sequences of *pfmsp*-*1* and *pfmsp*-*2* were analysed using EditSeq and SeqMan in the DNASTAR package (DNASTAR, Madison, WI, USA). The nucleotide sequences reported in this study have been deposited in the GenBank database under the accession numbers MH981972–MH982070 for *pfmsp*-*1*, and MH982071–MH982183 for *pfmsp*-*2*.

### Sequence analysis of *pfmsp*-*1* and *pfmsp*-*2* among the global *P. falciparum* population

The genetic diversity of *pfmsp*-*1* and *pfmsp*-*2* among global isolates was analysed. Accession numbers of sequences analysed in this study were presented in Additional file 1: Table S1. These sequences cover block 2 of *pfmsp*-*1*, and block 3 of *pfmsp*-*2*. The genetic polymorphism of each population was analysed by the methods as describe above.

### Recombination parameters and linkage disequilibrium

The recombination and linkage disequilibrium of Myanmar *pfmsp*-*1* and *pfmsp*-*2* were analysed. The recombination parameter (R), which contained the effective population size and probability of recombination between adjacent nucleotides per generation, and the minimum number of recombination events (Rm) were measured, using DnaSP ver. 5.10.00 [32]. Linkage disequilibrium (LD) between different polymorphic sites was computed in term of the R^2^ index, using DnaSP ver. 5.10.00. The R^2^ values were plotted against the nucleotide diversity distances with the two-tailed Fisher’s exact test of significance [32].

## Results

### Sequence polymorphism of Myanmar *pfmsp*-*1* block 2

**Fig. 2:**
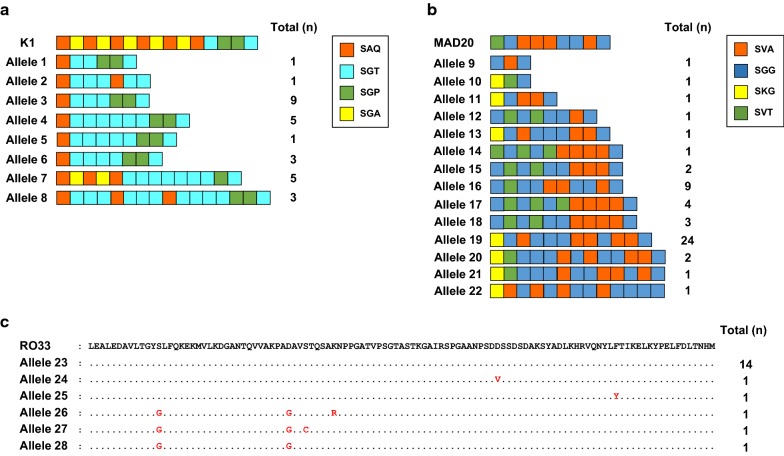
Schematic structures of the polymorphic patterns in Myanmar *pfmsp*-*1* block 2. A total of 28 different alleles, 8 alleles for K1 type (**a**), 14 alleles for MAD20 type (**b**), and 6 alleles for RO33 type (**c**), were identified in Myanmar *pfmsp*-*1* block 2. K1 type alleles differed in the number and arrangement of SAQ, SGT, SGP, and SGP motifs, meanwhile MAD20 type alleles varied in the number and ordering of SVA, SGG, SKG, and SVT motifs. The total number of isolates for each allele is indicated

Ninety-nine *pfmsp*-*1* block 2 were successfully amplified from 115 Myanmar *P. falciparum* isolates. They were classified into 3 different allele types: 28 sequences of K1 type, 52 sequences of MAD20 type, and 19 sequences of RO33 type. Amino acid sequence analysis of 99 Myanmar *pfmsp*-*1* revealed 28 different alleles (Fig. 2). K1 and MAD20 types were polymorphic, with different compositions of tripeptide repeat units (SAQ, SGT, SGA, and SGP for K1 type, and SVA, SGG, SKG, and SVT for MAD20 type). Meanwhile RO33 type sequences were less polymorphic, with only limited numbers of amino acid substitutions. In the K1 types, each allele had different numbers and arrangements of tripeptide repeat units (Fig. 2a). All 8 alleles (alleles 1 to 8) started with SAQ and terminated with SGT, but the middle tripeptide repeat units were highly polymorphic, with different numbers and arrangements of 3 tripeptide repeat units, SAQ, SGT, and SGP, in the alleles. The SGA tripeptide repeat unit was identified only in allele 7. The allele 3 was the most prevalent allele type, followed by alleles 4 and 7. The MAD20 types showed more diverse patterns of polymorphisms, with 14 different allele types, alleles 9 to 22. Most alleles were started by SGG or SKG, with the only exception of allele 14, which started with SVT; but all alleles were commonly terminated with SGG. The middle parts of the repeat region of MAD20 type alleles were also highly polymorphic, with different numbers and organizations of tripeptide repeat units, SVA, SGG, and SVT (Fig. 2b). Allele 19 was the most predominant MAD20-allele type, accounting for 46% of the 52 MAD20 sequences. A total of 6 alleles (alleles 23 to 28) were classified into RO33 type, and they had relatively well conserved sequences (Fig. 2c). The allele 23, which had identical sequences with RO33 sequence, was predominant (73.7%). Only 6 non-synonymous amino acid changes were identified in the other 5 alleles (alleles 24 to 28), but their frequencies were low (from 5.26 to 15.8%). The estimated MOI for Myanmar *pfmsp*-*1* was 1.98.

### Dynamic changes of Myanmar *pfmsp*-*1* alleles between 2004–2006 and 2013–2015

**Fig. 3:**
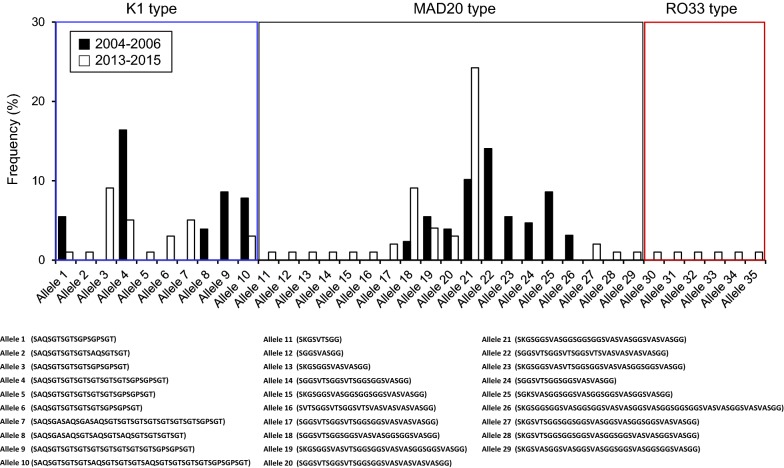
Dynamic changes of allele types of Myanmar *pfmsp*-*1* block 2 between 2004–2006 and 2013–2015

A total of 14 distinct alleles (5 alleles for K1 type, and 9 alleles for MAD20 type) of *pfmsp*-*1* were previously identified from 63 Myanmar *P. falciparum* isolates collected in 2004–2006 [26]. The genetic diversity of *pfmsp*-*1* analysed in this study was greater than that in the previous study. Although only K1 and MAD20 types were found in the previous years (2004–2006), all 3 types of *pfmsp*-*1*, K1, MAD20, and RO33, were identified in the recent years (2013–2015). The 7 alleles (alleles 1, 4, 10, 18, 19, 20, and 21) were commonly identified in both the previous and recent years. Interestingly, the overall frequencies of 5 alleles (alleles 1, 4, 10, 19, and 20) were decreased in recent years, compared to those in the previous years. Meanwhile, the frequencies of 2 alleles (alleles 18 and 21) were highly increased in the recent years than in the previous years. Whereas, 7 alleles (alleles 8, 9, and 22–26), which were found in the previous years, were not identified in the recent years. On the other hand, 21 alleles (alleles 2, 3, 5–7, 11–17, and 27–35) were observed only in the recent years, but not in the previous years (Fig. 3).

### Population structure of *pfmsp*-*1* in global *P. falciparum* isolates

**Fig. 4:**
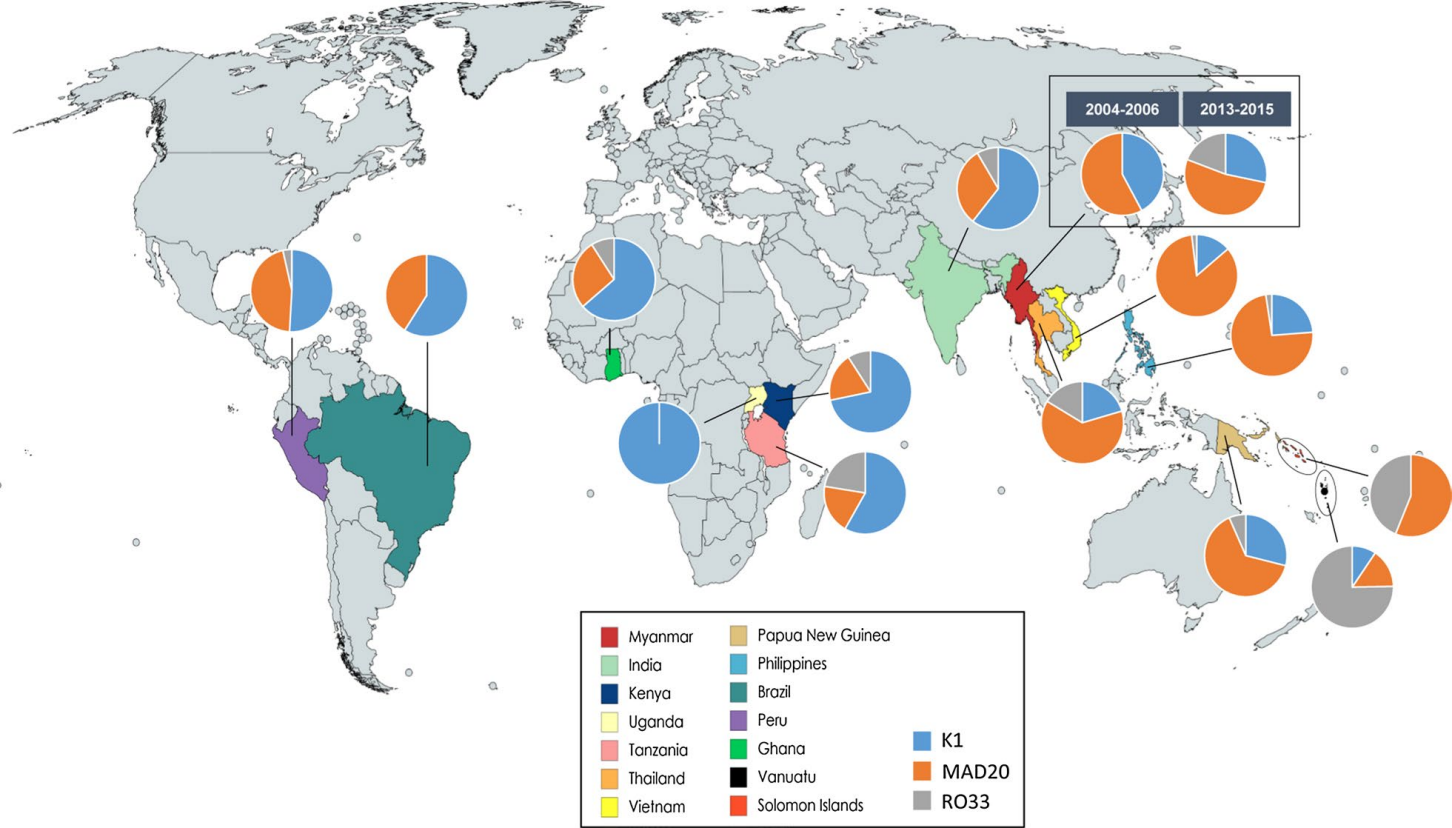
Geographical distribution and allelic diversity of *pfmsp*-*1* in global isolates. Global *pfmsp*-*1* sequences were obtained from GenBank. Each country is marked with a different colour. The pie chart represents the percentage of *pfmsp*-*1* family types (K1, MAD20, and RO33) in each country

The *pfmsp*-*1* sequences from different geographic areas of Myanmar, India, Thailand, Vietnam, Philippines, Papua New Guinea, Solomon Islands, Vanuatu, Ghana, Kenya, Tanzania, Uganda, Peru, and Brazil were comparatively analysed, to understand the genetic structure of *pfmsp*-*1* in the global *P. falciparum* population. Although the K1 and MAD20 types were the dominant ones in global *pfmsp*-*1*, the overall prevalence and distribution of *pfmsp*-*1* allelic types differed by country or continent (Fig. 4). The main allelic type in Asian countries was MAD20, accounting for more than 52% for each country. Indian isolates were the only exception, which showed greater prevalence of K1 type (60.6%) than MAD20 type (31.0%). Similar to Asian countries, MAD20 was also the predominant allelic type in the Pacific *pfmsp*-*1*: 64.5% in Papua New Guinea, and 56.1% in the Solomon Islands. However, the major allelic type in Vanuatu was RO33 type, which comprised approximately 75.3%. Meanwhile, higher frequencies of K1 allelic types were found in the African *pfmsp*-*1* (Ghana, 63.6%; Kenya, 71.7%; Tanzania, 58.1%; Uganda, 100%) and the South American *pfmsp*-*1* (Brazil, 58.9%; Peru, 50.8%). Comparative analysis of 1043 global *pfmsp*-*1* sequences revealed that a total of 267 distinct alleles (152 for K1 type, 97 for MAD20 type, and 18 for RO33 type) were found in the global *pfmsp*-*1*. The block 2 region of all alleles belonging to K1 type consistently started with SAQ, and terminated with SGT or SGP (Additional file 2: Table S2). Several amino acid substitutions were observed in India (SAQ to SGQ/SAP/SAL/SAT/SAR, and SGT to SVT), Kenya (SAQ to GAQ/SVQ), and Tanzania (SGA to NGA, and SGT to SGS), but their frequencies were very low. Some alleles were shared by different countries in different proportions, although most alleles were observed in a country-specific manner. For the MAD20 type, 97 alleles were found in the global *pfmsp*-*1*. Most alleles showed restricted distribution patterns within the same continent, and a large numbers of alleles were country-specific (Additional file 3: Table S3). A total of 18 distinct alleles classified into RO33 type were identified in the global *pfmsp*-*1*. Thirteen di-morphic and 2 tri-morphic amino acid changes were found at 15 positions, in which D67G was the most common amino acid change, with a frequency of 59.1% (Additional file 4: Figure S1a). Allele 3 was the dominant allelic type, which was shared by Asian and Pacific countries (Additional file 4: Figure S1b). Menawhile, alleles 1, 7, 8, 9, 10, 11, 12 and 14 were identified only in Africa countries.

### Sequence polymorphism of *pfmsp*-*2* block 3 in Myanmar *P. falciparum* isolates

**Fig. 5:**
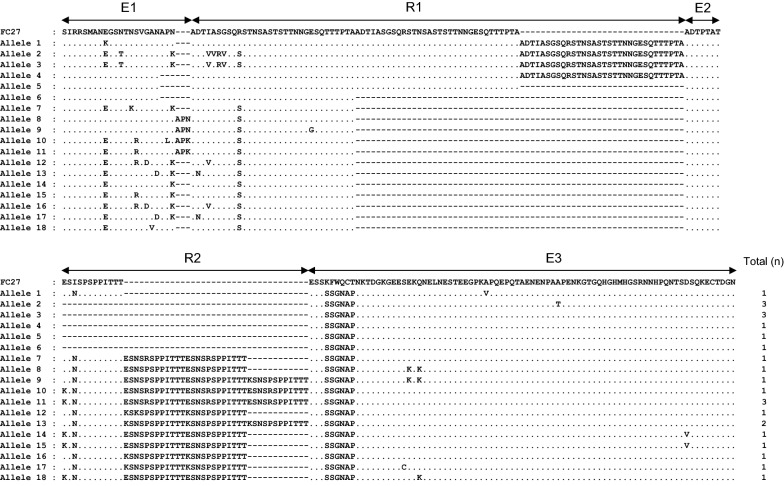
Sequence polymorphisms of FC27 allelic types of Myanmar *pfmsp*-*2*. The 18 alleles of FC27 type were identified in Myanmar *pfmsp*-*2*. The central variable regions of allelic types are compared to the reference sequence FC27 (J03828). Identical residues are indicated by dots. Dashes represent gaps introduced to maximize the alignment. The family-specific regions (E1–E3) and the two tandem repeat regions (R1 and R2) are indicated

**Fig. 6:**
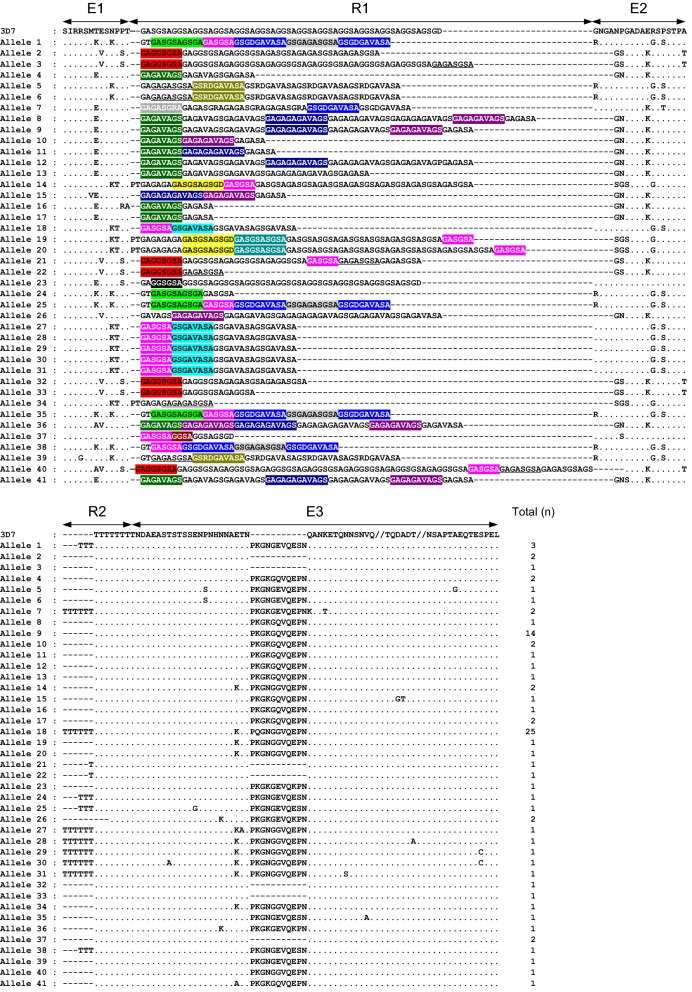
Sequence polymorphisms of 3D7 allelic types of Myanmar *pfmsp*-*2* block 3. The 41 alleles of 3D7 type were identified in Myanmar *pfmsp*-*2*. The central variable regions of allelic types are compared to the reference sequence 3D7 (X53832). Identical residues are indicated by dots. Dashes represent gaps introduced to maximize the alignment. The family-specific regions (E1–E3) and the two tandem repeat regions (R1 and R2) are indicated. Each repeat unit in R1 region is shaded by different colors, or represented by underline. The total number of each allele is indicated

One hundred and thirteen *pfmsp*-*2* block 3 were successfully amplified from 115 Myanmar *P. falciparum* isolates. Sequence analysis of the 113 Myanmar *pfmsp*-*2* sequences showed that 25 sequences were classified into the FC27 type, while the other 88 sequences belonged to the 3D7 type. Both FC27 and 3D7 types showed remarkably different dimorphic structures in the variable central region. The 25 FC27 type sequences were grouped into 18 distinguishable alleles in amino acid level (Fig. 5). The FC27 type alleles had different numbers of repetitive R1 (96 bp) and R2 (36 bp) regions. The R1 region was comprised 1–3 copies of the 96 bp family specific-motif, which encoded ADTIASGSQSSTNSASTSTTNNGESQTTTPTA or its variants. Amino acid changes at 7 positions were found in the R1 region. All amino acid polymorphisms were located at the first copy of R1 with the highest frequency of R72S (85.7%), but the subsequent 2 copies were well conserved in all Myanmar FC27 type *pfmsp*-*2* sequences. The E2 repeat unit of 21 bp was well conserved in all alleles. The R2 region presented highly polymorphic nature with varying numbers of 36 bp-repeat copies, ranged from 0 to 4 copies. A typical non-synonymous substitution of 6 amino acids (SSGNAP) was found at the E3 region in all FC27 allelic types of Myanmar *pfmsp*-*2*. Several non-synonymous amino acid changes were also identified in E1, R2, and E3, but their frequencies were very low. The 3D7 allelic type sequences showed more complicated polymorphic characters than FC27 type sequences (Fig. 6). They were grouped into 41 distinct alleles in amino acid level. Multiple copies of repeat units associated with a large numbers of flanking regions were identified in R1. The differences in arrangement, length, and number of repeat units contributed to the immense polymorphic character of the R1 region. The R2 poly-threonine repeat, which is typical of the 3D7 family type, also varied among the alleles with the number of repeats of threonine residues, ranged from 5 to 14. Duplication of PT motif at the 3′ end of E1 was found in 4 alleles (alleles 14, 19, 20, and 34). The insertion of 11 amino acids, PKGNGEVQESN/PKGKGQVQEPN/PKGNGEVQEPN or PKGKGEVQKPN, in E3 was identified in most alleles except 7 alleles (alleles 2, 3, 21, 22, 32, 33, and 37). A total of 19 di-morphic, 4 tri-morphic, and 2 polymorphic amino acid changes were also found in E1, E2, and E3 regions, which were responsible for the extreme diversity of 3D7 type alleles, compared with the FC27 type alleles in the Myanmar *pfmsp*-*2*. The estimated MOI for Myanmar *pfmsp*-*2* was 2.41.

### Comparison of genetic diversity of R1 and R2 regions in the global *pfmsp*-*2*

**Fig. 7:**
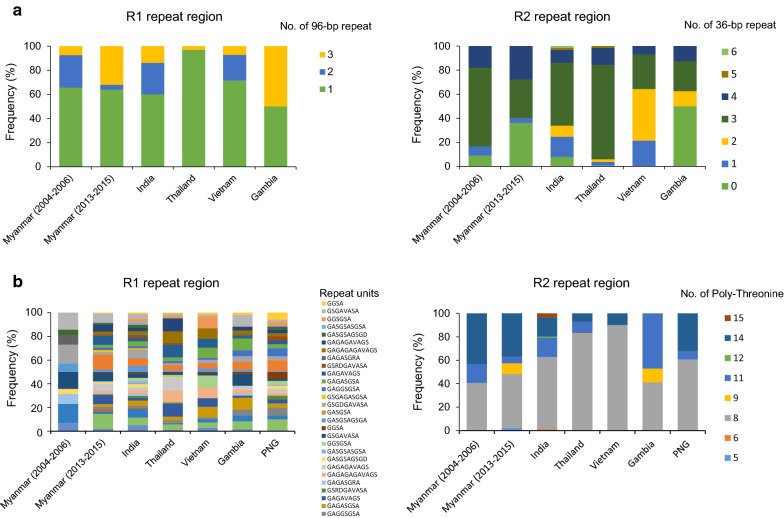
Comparisons of R1 and R2 repeat regions in global *pfmsp*-*2* block 3. The frequency of number of region specific-repeat units was comparatively analysed in FC27 (**a**) and 3D7 (**b**) types of Myanmar and global *pfmsp*-*2*

The genetic diversity of R1 and R2 regions in the global *pfmsp*-*2* were comparatively analysed (Fig. 7a). For the R1 region of FC27 family type, Myanmar, India, and Vietnam *pfmsp*-*2* had (1 to 3) copies of 96-bp repeat, while Thailand and Gambia *pfmsp*-*2* had either 1 or 3 copies. The frequency of single copy of 96-bp repeat was the highest in all populations except Gambia, but the frequencies of other copy numbers differed by country. The numbers of R2 specific 36-bp repeat in all analysed global *pfmsp*-*2* sequences ranged from 0 to 6 copies, in which 3 copies were predominant in Myanmar (2004–2006) (65.7%), India (52.3%), and Thailand (78.3%). Interestingly, the frequency of sequences with non 36-bp repeat was high in Myanmar (2013–2015) (36%) and Gambia (50%). Five and 6 copies of the 36-bp repeat were found in only Thailand and India, but the frequencies were very low in both countries. Comparative analysis of repeat units in R1 region of 3D7 type in global *pfmsp*-*2* displayed a very complicated pattern of diversity (Fig. 7b). The repeat numbers and the distributions of repeat units varied among the populations. In the cases of Myanmar (2013–2015), India, Thailand, and Papua New Guinea, the most abundant repeat types contained the duplication/variations of GA or SG dipeptides. The GSGDGAVASA, GASGSAGSGA, and GSGAVASA were found in all countries with different frequencies, followed by GGSA, GASGSA, GAGGSGSA, GAGASGSA, GAGAVAGS, and GAGAGAGAVAGS. The frequency of each repeat type in each country was also extremely complicated in the R1 region. The number of threonine in the R2 region varied ranged from 5 to 15. Overall, the India, Thailand, Vietnam, and Papua New Guinea *pfmsp*-*2* had lower numbers of poly-threonine than those of the other countries.

### Linkage disequilibrium of Myanmar *pfmsp*-*1* and *pfmsp*-*2* between 2004–2006 and 2013–2015

**Fig. 8:**
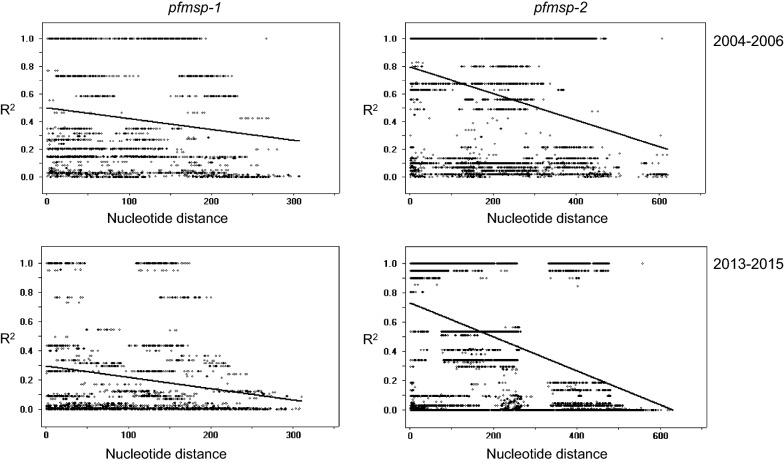
The linkage disequilibrium (LD) plot of Myanmar *pfmsp*-*1* and *pfmsp*-*2* between 2004–2006 and 2013–2015. The LD plot showing non-random associations between nucleotide variations in Myanmar *pfmsp*-*1* and *pfmsp*-*2* during two periods at different polymorphic sites. The R^2^ values are plotted against the nucleotide distances with two-tailed Fisher’s exact test of significance

The values for minimum number of recombination events between adjacent polymorphic sites (Rm) were 4 and 7 for Myanmar *pfmsp*-*1* and *pfmsp*-*2* in *P. falciparum* collected in 2004–2006, respectively. The Rm values were greater in Myanmar *P. falciparum* collected in 2013–2015 (*pfmsp*-*1*: 7; *pfmsp*-*2*: 9) (Table 1). The LD index R^2^ plot for the Myanmar *pfmsp*-*1* and *pfmsp*-*2* populations in both the periods were declined across the analysed regions, indicating that intragenic recombination could be contributing to the genetic diversity of the Myanmar *pfmsp*-*1* and *pfmsp*-*2* populations (Fig. 8). The reduction rate of LD index R^2^ was greater in Myanmar *pfmsp*-*1* and *pfmsp*-*2* in 2013–2015, compared to those in 2004–2006.

**Table 1:** Recombinant events in Myanmar *pfmsp*-*1* and *pfmsp*-*2* between 2004–2006 and 2013–2015

	Years	n	Ra	Rb	Rm
PfMSP-1	2004–2006	63	0.0046	1.7	4
	2013–2015	63	0.0134	4.9	7
PfMSP-2	2004–2006	99	0.0000	0.001	7
	2013–2015	113	0.0000	0.001	9

## Discussion

The genetic structure of *P. falciparum* population plays a very important role in the natural acquisition of immunity against malaria infections [33, 34]. Therefore, knowledge of the genetic nature and structure of the *P. falciparum* population is important to developing strategies to control the disease, including the design of effective vaccines against *P. falciparum*. Due to their polymorphic features, *pfmsp*-*1* and *pfmsp*-*2* have been employed as polymorphic markers in studies for malaria transmission dynamics in the natural *P. falciparum* population [21, 24, 35–39]. *Plasmodium falciparum* cases in Myanmar have decreased in recent years [30], but this species is still the most critical priority for malaria control and prevention in the country. In this study, the polymorphic characteristics of *pfmsp*-*1* and *pfmsp*-*2* were analysed in Myanmar *P. falciparum* isolates, to gain in-depth insight into the genetic nature of the Myanmar *P. falciparum* population.

High levels of genetic polymorphisms were identified in the Myanmar *pfmsp*-*1* and *pfmsp*-*2*. Sequence analysis of 99 *pfmsp*-*1* block 2 sequences revealed that a total of 28 distinct alleles (8 for K1 type, 14 for MAD20 type, and 6 for RO33 type) were identified in the Myanmar *P. falciparum* population that was analysed in this study. Similar to previous studies, different numbers and arrangements of unique tripeptide repeats were the main factors to contribute to the allelic diversity in the K1 and MAD20 types of Myanmar *pfmsp*-*1* [15, 26]. In the case of *pfmsp*-*2*, 113 Myanmar sequences were classified into FC27 and 3D7 types, which showed highly different dimorphic structures in the variable central regions. The alleles of Myanmar *pfmsp*-*2* belonging to the FC27 family were polymorphic, which was characterized by various numbers of FC27 family specific repeats in the R1 and R2 regions, in accord with previous reports [24, 40]. The 3D7 type showed more extensive sequence diversity. Besides the major polymorphic characters in the R1 and R2 variable regions, non-synonymous amino acid changes were also found in family specific regions (E1, E2, and E3) of 3D7 type alleles, which make the genetic diversity of 3D7 type alleles much greater than that of FC27 type alleles. Interestingly, the overall genetic diversity of Myanmar *pfmsp*-*1* and *pfmsp*-*2* increased in recent years (2013–2015) compared with the previous years (2004–2006), even though the incidence of malaria in the studied areas has reduced in recent years [30]. A total of 14 distinct alleles (5 alleles for K1 type, and 9 alleles for MAD20 type) of *pfmsp*-*1* were previously identified from 63 Myanmar *P. falciparum* isolates collected in 2004–2006 [26]. Meanwhile, 28 alleles for all 3 types of *pfmsp*-*1*, K1, MAD20, and RO33, were identified in 99 sequences in recent years (2013–2015). Moreover, comparative analysis of alleles belonging to K1 and MAD20 types in the previous and recent years revealed that they have changed remarkably in recent years, with the appearance of new alleles, and disappearance of pre-existing alleles. The *pfmsp*-*2* also showed a similar pattern of genetic diversity to *pfmsp*-*1* in the Myanmar *P. falciparum* population. Twenty-two different alleles (7 for FC27 type, and 15 for 3D7 type) in 148 sequences were identified in previous years, but 59 alleles (18 for FC27, and 41 for 3D7) were identified in 113 sequences in recent years. As the numbers of *P. falciparum* isolates analysed both in previous and recent years were limited, it may not be easy to insist that the overall genetic diversity of Myanmar *pfmsp*-*1* and *pfmsp*-*2* has increased in recent years, compared to previous years. However, considering that the number of alleles for Myanmar *pfmsp*-*1* and *pfmsp*-*2* over the total number of sequences analysed has increased in recent years compared with those of previous years, it would be plausible to propose that the genetic diversity of the Myanmar *pfmsp*-*1* and *pfmsp*-*2* in the *P. falciparum* population in the studied areas has increased recently, and high levels of genetic complexity are still maintained in the population. High levels of the genetic diversity of *pfmsp*-*1* and *pfmsp*-*2* in the recent *P. falciparum* population in Southeast and Western Myanmar have also been reported [36].

It is not clear why the Myanmar *pfmsp*-*1* and *pfmsp*-*2* population structure in the studied areas has diversified so drastically in recent years, compared to the previous years, 2004–2006, despite the remarkable reduction of transmission in the last decade. In a declining population, rare alleles usually disappear, and the probability of multiplicity of infection (MOI) is reduced, so inbreeding is likely to increase. Overall decline of MOI was also identified in the Myanmar *P. falciparum* population analysed in this study. These declining patterns have been identified in both the *P. falciparum* and *P. vivax* populations, even in low transmission areas and pre-elimination settings [41–45]. This could be explained by asymptomatic carriers acting as fundamental reservoirs contributing to malaria transmission. Indeed, a substantial level of asymptomatic infections in the studied areas has been reported [30]. Asymptomatic patients can facilitate superinfection, and the genotypes infected in asymptomatic patients may contribute to the maintenance or generation of genetic complexity of the Myanmar *P. falciparum* population. Another plausible explanation for the increased genetic diversity of Myanmar *pfmsp*-*1* and *pfmsp*-*2* in recent years can be speculated as the higher values of Rm for both genes, compared to those of previous years. The high values of recombination parameters identified in the recent *pfmsp*-*1* and *pfmsp*-*2* suggest that high levels of meiotic recombination events may occur in the genes, which render the genetic make-up of the genes more complex. The finding that high levels of MOI comparable to previous years (2.03 for *pfmsp*-*1* and 2.35 for *pfmsp*-*2*) were still maintained in recent years (1.98 for *pfmsp*-*1* and 2.41 for *pfmsp*-*2*) also supported this notion. Further combined analyses of other polymorphic markers, such as circumsporozoite protein and apical membrane antigen-1, with larger number of isolates, may be necessary for in-depth understanding of the genetic nature and genetic flow of the Myanmar *P. falciparum* population.

The comparative population structural analysis of *pfmsp*-*1* and *pfmsp*-*2* in the global *P. falciparum* population also suggests that high levels of genetic diversity of the two genes are maintained in the global *P. falciparum* population. Interestingly, the overall distribution of allele types of *pfmsp*-*1* differed by geographical origin. Although K1 and MAD20 types were largely predominant in the global *pfmsp*-*1*, the overall prevalence and distribution of *pfmsp*-*1* allelic types differed by country or continent. In Southeast Asia countries, MAD20 was the most prevalent type, followed by K1 type. The MAD20 type was also dominant in Pacific countries, but RO33 allele type was also identified as of high proportion in these countries. Meanwhile, K1 type was the most prevalent allele type in the South American and African *pfmsp*-*1*. Further analysis of the global *pfmsp*-*1* sequences showed more complicated patterns of genetic diversity of the global *pfmsp*-*1*. Most *pfmsp*-*1* alleles among the total 267 global *pfmsp*-*1* alleles were identified in a country-specific manner. The genetic diversity of K1 and MAD20 types was much greater than that of RO33 type. The *pfmsp*-*2* also showed high level of genetic diversity in the global *pfmsp*-*2* population. The R1 and R2 regions were highly polymorphic, with different numbers and arrangements of repeat units. The geographic patterns of genetic differentiation suggest that the functional consequences of the polymorphism should be considered for designing a vaccine based on *pfmsp*-*1* or *pfmsp*-*2*. The results of this study contribute insight into the genetic nature of Myanmar *pfmsp*-*1* and *pfmsp*-*2*, as well as aid understanding of the genetic diversity of the global *pfmsp*-*1* and *pfmsp*-*2*, and may provide valuable information for the development of an effective vaccine based on *pfmsp*-*1* and *pfmsp*-*2*.

## Conclusion

The Myanmar *pfmsp*-*1* and *pfmsp*-*2* populations showed high levels of genetic diversity, and have remarkably diversified in recent years, despite the rapid decline of malaria cases in the country for the last decade. These suggest that the Myanmar *P. falciparum* population still remains of sufficient size to allow the generation and maintenance of genetic diversity. Therefore, continuous molecular epidemiological surveillance to supervise the genetic variation of the parasite in Myanmar would be necessary. Additional studies to examine the dynamics of the genetic diversity and gene flow of the *P. falciparum* population combined with factors including the malaria transmission intensity based on entomological inoculation rates, and the immune status of *P. falciparum*-infected individuals, may contribute to the guidance of malaria interventions. The extreme genetic diversity patterns of *pfmsp*-*1* and *pfmsp*-*2* found in the global *P. falciparum* population also warrant continuous monitoring of the genetic diversity of the two genes in the global population, to better understand the polymorphic nature and evolutionary aspect of the vaccine candidate antigens in the global *P. falciparum* population.

## Additional files

**Additional file 1: Table S1.** Global *pfmsp*-*1* and *pfmsp*-*2* sequences analysed in this study.

**Additional file 2: Table S2.** Distribution of K1 alleles among global *pfmsp-1*.

**Additional file 3: Table S3.** Distribution of MAD20 alleles among global *pfmsp-1*.

**Additional file 4: Fig. S1.** Polymorphisms and distributions of RO33 alleles among global *pfmsp-1*. (a) Sequence alignment of RO33 allelic types among global *pfmsp-1*. The eighteen RO33 alleles were identified in global *pfmsp-1*. The dots represent residues identical to the reference sequence of RO33 (AB276005). Amino acid changes were marked with reds. (b) Frequency of RO33 alleles among global *pfmsp-1*. PNG, Papua New Guinea.

## Data Availability

The data supporting the conclusions of this article are provided within the article and its additional file. The original datasets analysed in the current study are available from the corresponding author upon request. The newly generated sequences were deposited in the GenBank database under the Accession Number MH981972–MH982070 for *pfmsp*-*1* and MH982071– MH982183 for *pfmsp*-*2*.

## Abbreviations

ACT: artemisinin-based combination therapy
GMS: Greater Mekong Subregion
LD: linkage disequilibrium
MOI: multiplicity of infection
PCR: polymerase chain reaction (PCR)
PfMSP-1: merozoite surface protein-1 of *Plasmodium falciparum*
PfMSP-2: merozoite surface protein-2 of *Plasmodium falciparum*
PNG: Papua New Guinea
R: recombination parameter
Ra: recombination parameter between adjacent sites
Rb: recombination parameter for entire gene
Rm: minimum number of recombination events between adjacent polymorphic sites
rRNA: 18S ribosomal RNA
UV: ultraviolet
