# Supplementary material for: Changing pattern of the genetic diversities of Plasmodium falciparum merozoite surface protein-1 and merozoite surface protein-2 in Myanmar isolates
Source: Malar J. 2019 Jul 16;18:241. doi: 10.1186/s12936-019-2879-7 (PMC6636015; doi:10.1186/s12936-019-2879-7)
Supplement: Supplementary file 1 — Additional file 1: Table S1. Global pfmsp-1 and pfmsp-2 sequences analysed in this study. [file 12936_2019_2879_MOESM1_ESM.docx]

**Table S1. Global *pfmsp-1* and *pfmsp-2* sequences analysed in this study**

| Country | PfMSP-1 | PfMSP-2 |
| --- | --- | --- |
| Myanmar  (2004-2006) | EU445555 – EU445566,  GQ861442 – GQ861445 (n = 16) | EU647447 – EU647468  (n = 22) |
| Thailand | AB502546 – AB502586,  AB276707 – AB276708,  AB276710 – AB276711, AB276713, AB276715 –AB276716, AB276718 (n = 49) | JX885898 – JX885980, GQ890790 – GQ890871, EU810410 – EU810640  (n = 360) |
| India | MF772523 – MF772552, HM568565 – HM568602,  JF460905 – JF460923,  KY425905 – KY425959 (n = 142) | KY425960 – KY426009, HM568603 – HM568668,  JX283501 – JX283537  (n = 153) |
| Vietnam | AF509635 , AF509641 – AF509654, AF509669 – AF509703, AF509706 (n = 51) | AF-104684 – AF104717  (n = 34) |
| Philippines | AB502587 – AB502628 (n = 42) |  |
| Brazil | AF509630 – AF509634,  AF509636 – AF509640,  AF509655 – AF509668, AF509701, AF509705, AF509707 – AF509719 (n = 39) |  |
| Peru | FJ612009 – FJ612065 (n = 57) |  |
| Uganda | JX416338 – JX416341 (n = 4) |  |
| Kenya | MG675458 – MG675556 (n = 99) |  |
| Gambia |  | U91650, U91652 – U91677  (n = 27) |
| Tanzania | AB502443 – AB502513  AF061119–AF061141, AF061148–AF061151 (n = 98) |  |
| Ghana | AB502514 – AB502545 (n = 33) |  |
| Papua New Guinea | AB502629 – AB502704 (n = 76) | U07001, U07009, U16840, U16842, AY534507, DQ162662, DQ185319 – DQ185320, AJ318752 – AJ318755, DQ166534 – DQ166545, DQ158904, DQ168571 – DQ168572, DQ174442, DQ171731 – DQ171732, M73810 (n = 32) |
| Vanuatu | AB715435 – AB715519 (n = 85) |  |
| Solomon Islands | AB502705 – AB502745 (n = 41) |  |
