# Supplementary material for: Changing pattern of the genetic diversities of Plasmodium falciparum merozoite surface protein-1 and merozoite surface protein-2 in Myanmar isolates
Source: Malar J. 2019 Jul 16;18:241. doi: 10.1186/s12936-019-2879-7 (PMC6636015; doi:10.1186/s12936-019-2879-7)
Supplement: Supplementary file 2 — Additional file 2: Table S2. Distribution of K1 alleles among global pfmsp-1. [file 12936_2019_2879_MOESM2_ESM.docx]

**Table S2. Distribution of K1 alleles among global pfmsp-1**

| No. | Alleles | Myanmar (04-06) | Myanmar (13-15) | India | Thailand | Vietnam | Philippines | Kenya | Uganda | Tanzania | Ghana | PNG | Vanuatu | Brazil | Peru |
| --- | --- | --- | --- | --- | --- | --- | --- | --- | --- | --- | --- | --- | --- | --- | --- |
| 1 | SAQSGTSGTSGPSGPSGT | 7 | 1 | 1 |  |  |  |  |  |  |  |  |  |  |  |
| 2 | SAQSGTSGTSGTSAQSGTSGT |  | 1 | 4 |  |  |  |  |  |  |  |  |  |  |  |
| 3 | SAQSGTSGTSGTSGPSGPSGT |  | 9 | 4 |  | 1 | 5 |  |  | 2 |  |  |  |  |  |
| 4 | SAQSGTSGTSGTSGTSGTSGTSGPSGPSGT | 21 | 5 | 3 | 5 | 2 |  |  |  |  |  |  | 8 |  |  |
| 5 | SAQSGTSGTSGTSGTSGTSGPSGPSGT |  | 1 |  |  | 2 | 5 |  |  | 1 |  |  |  | 1 |  |
| 6 | SAQSGTSGTSGTSGTSGPSGPSGT |  | 3 | 3 |  | 2 |  |  |  |  |  |  |  | 1 |  |
| 7 | SAQSGASAQSGASAQSGTSGTSGTSGTSGTSGTSGTSGPSGT |  | 5 |  |  |  |  |  |  |  |  |  |  |  |  |
| 8 | SAQSGTSGTSGTSAQSGTSGTSGTSAQSGTSGTSGTSGTSGPSGPSGT | 10 | 3 | 2 |  |  |  |  |  |  | 1 |  |  |  |  |
| 9 | SAQSGTSAQSGTSGTSGTSGTSGPSGPSGP |  |  |  |  |  |  |  |  |  | 1 |  |  |  |  |
| 10 | SAQSGASAQSGASAQSGTSGTSGT |  |  |  |  |  |  |  |  |  | 1 |  |  |  |  |
| 11 | SAQSGASAQSGASAQSGASAQSGTSGPSGPSGT |  |  | 1 | 1 |  |  | 1 |  | 2 | 3 |  |  |  | 11 |
| 12 | SAQSGASAQSGASAQSGTSGPSGPSGT |  |  |  |  |  |  | 1 |  | 1 | 3 |  |  |  |  |
| 13 | SAQSGASAQSGTSAQSGASAQSGTSGTSGPSGPSGT |  |  |  |  |  |  |  |  |  | 1 |  |  |  |  |
| 14 | SAQSGASAQSGASAQSGPSGPSGT |  |  |  |  |  |  |  |  |  | 2 |  |  |  |  |
| 15 | SAQSGASAQSGASAQSGTSGPSGP |  |  |  |  |  |  |  |  |  | 1 |  |  |  |  |
| 16 | SAQSGASAQSGTSGPSGPSGT |  |  |  |  |  |  |  |  |  | 1 |  |  |  |  |
| 17 | SAQSGASAQSGASAQSGASAQSGTSGPSGPSGPSGT |  |  |  |  |  |  | 1 |  |  | 2 |  |  | 1 |  |
| 18 | SAQSGASAQSGASAQSGASAQSGASAQSGTSGPSGPSGT |  |  | 4 |  |  |  | 1 |  | 3 | 1 |  |  |  |  |
| 19 | SAQSGTSGTSGPSGTSGPSGTSGPSGTSGPSGTSGPSGPSGT |  |  |  |  |  |  | 1 |  |  | 1 |  |  |  |  |
| 20 | SAQSGASAQSGTSGPSGTSGPSGTSGPSGTSGPSGTSGPSGPSGT |  |  |  |  |  |  |  |  |  | 1 |  |  |  |  |
| 21 | SAQSGTSGTSGTSGTSGTSGTSGTSGTSAQSGTSAQSGTSGTSGTSGPSGT |  |  |  |  |  |  |  |  |  | 1 |  |  |  |  |
| 22 | SAQSGTSGTSGTSGTSGTSGTSGTSGPSGPSGT |  |  | 2 |  |  |  | 1 |  |  |  | 1 |  |  |  |
| 23 | SAQSGTSGTSGTSGTSGTSGTSGTSGTSGPSGPSGT | 11 |  | 2 |  |  |  | 2 |  | 1 |  |  |  |  |  |
| 24 | SAQSGTSGTSGTSAQSGTSGTSAQSGTSGTSGTSGPSGPSGT |  |  | 1 |  |  |  |  |  |  |  |  |  |  |  |
| 25 | SAQSGTSGTSGTSAQSGTSGTSGTSGTSGPSGTSGTSGTSGPSGPSGPSGT |  |  | 1 |  |  |  |  |  |  |  |  |  |  |  |
| 26 | SAQSGTSGPSGPSGT |  |  | 2 |  |  |  |  |  |  |  |  |  |  |  |
| 27 | SAQSGTSGTSGTSAQSGTSGTSAQSGTSGTSGTSGTSGTSGPSGPSGT |  |  | 2 |  |  |  |  |  |  |  |  |  |  |  |
| 28 | SAQSGTSGTSGTSAQSGTSGTSAQSGTSGTSGTSGTSGPSGPSGT |  |  | 1 |  |  |  |  |  |  |  |  |  |  |  |
| 29 | SAQSGTSGTSGPSGTSGPSGTSGPSGT |  |  | 1 |  |  |  | 1 |  | 1 |  |  |  |  |  |
| 30 | SAQSGTSGTSGTSGTSGTSGTSGTSGTSGTSGTSGTSGPSGPSGPSGPSGT |  |  | 1 |  |  |  |  |  |  |  |  |  |  |  |
| 31 | SAQSGTSGTSGTSGTSGTSGTSGTSGTSGTSGTSGPSGPSGT |  |  | 1 |  |  |  |  |  | 1 |  | 7 |  | 4 | 18 |
| 32 | SAQSGTSGTSGTSGTSGTSGTSGTSGTSGTSGPSGPSGPSGPSGT |  |  | 1 |  |  |  | 1 |  |  |  |  |  |  |  |
| 33 | SAQSGTSGTSGTSGTSGTSGTSGPSGPSGPSGT |  |  | 1 |  |  |  |  |  | 1 |  |  |  |  |  |
| 34 | SAQSGTSGTSAQSGTSAQSGTSGTSGTSGPSGPSGPSGPSGT |  |  | 1 |  |  |  |  |  |  |  |  |  |  |  |
| 35 | SAQSGASAQSGTSGPSGPSGPSGT |  |  | 1 |  |  |  |  |  |  |  |  |  |  |  |
| 36 | SAQSGTSGTSGTSGTSGPSGPSGPSGT |  |  | 1 |  |  |  | 1 |  |  |  |  |  |  |  |
| 37 | SAQSGASGPSGASGQSGPSGPSGTSGPSGPSGT |  |  | 1 |  |  |  |  |  |  |  |  |  |  |  |
| 38 | SAQSGTSGTSGTSAQSGTSGTSGTSAQSGTSGTSGTSGTSGPSGPSGPSGP |  |  | 2 |  |  |  |  |  |  |  |  |  |  |  |
| 39 | SAQSGTSGTSGTSGTSGTSGTSGPSGPSGPSGPSGT |  |  | 1 |  |  |  |  |  |  |  |  |  |  |  |
| 40 | SAQSGTSGTSAQSGTSGTSAQSGTSGTSGTSGPSGPSGT |  |  | 1 |  |  |  |  |  |  |  |  |  |  |  |
| 41 | SAQSGTSGTSGTSGTSGPSGPSGPSGTSGTSGTSGPSGPSGPSGT |  |  | 1 |  |  |  |  |  |  |  |  |  |  |  |
| 42 | SAQSGTSGTSAPSGTSGTSGTSGTSGTSGTSGTSGPSGT |  |  | 1 |  |  |  |  |  |  |  |  |  |  |  |
| 43 | SAQSGTSGTSGTSAQSGTSGTSAQSGTSGTSGTSGTSGPSGPSGPSGPSGP |  |  | 1 |  |  |  |  |  |  |  |  |  |  |  |
| 44 | SAQSVTSGTSGTSAQSGTSGTSGTSAQSGTSGTSGTSGTSGPSGPSGPSGT |  |  | 1 |  |  |  |  |  |  |  |  |  |  |  |
| 45 | SAQSVTSGTSGTSAQSGTSGTSAQSGTSGTSGTSGTSGTSGPSGPSGT |  |  | 1 |  |  |  |  |  |  |  |  |  |  |  |
| 46 | SAQSGTSGTSAQSGTSGTSGTSGTSGTSGPSGPSGT |  |  | 1 | 1 |  |  |  |  |  |  |  |  |  |  |
| 47 | SAQSVTSGTSGTSAQSGTSGTSAQSGTSGTSGTSGTSGT |  |  | 1 |  |  |  |  |  |  |  |  |  |  |  |
| 48 | SAQSGTSGTSGTSGTSGTSGPSGPSGPSGT |  |  | 1 |  |  |  |  |  |  |  |  |  |  |  |
| 49 | SAQSGTSGTSGTSGTSGTSGPSGPSGPSGPSGT |  |  | 1 |  |  |  |  |  |  |  |  |  |  |  |
| 50 | SAQSGTSGTSGTSAQSGTSGTSAQSGTSGTSGTSGTSGPSGPSGPSGPSGPSGT |  |  | 1 |  |  |  |  |  |  |  |  |  |  |  |
| 51 | SAQSGTSGTSGTSGTSGPSGPSGPSGPSGT |  |  | 1 |  |  |  |  |  |  |  |  |  |  |  |
| 52 | SAQSGASGTSGASALSATSGPSGPSGT |  |  | 1 |  |  |  |  |  |  |  |  |  |  |  |
| 53 | SAQSGTSAQSGTSGTSGTSGTSGTSGPSGPSGT |  |  | 1 |  |  |  |  |  |  |  |  |  |  |  |
| 54 | SAQSGTSGTSGTSAQSGTSGTSGTSGTSGTSGPSGPSGT |  |  | 1 |  |  |  |  |  |  |  |  |  |  |  |
| 55 | SAQSGTSGTSGTSGTSGTSGTSGTSGTSGTSGPSGPSGT |  |  | 1 | 3 |  |  |  |  |  |  | 2 |  |  |  |
| 56 | SAQSGTSGTSGPSGTSGPSGT |  |  | 1 |  |  |  |  |  |  |  |  |  |  |  |
| 57 | SAQSGASAQSGASAQSGASAQSGASAQSGASAQSGASAQSGTSGPSGPSGT |  |  | 7 |  |  |  |  |  |  | 1 |  |  |  |  |
| 58 | SAQSGASAQSGASAQSGASAQSGTSGTSGPSGPSGT |  |  | 6 |  |  |  |  |  |  |  |  |  |  |  |
| 59 | SAQSGASAQSGASAQSGASAQSGASAQSGASAQSGTSGPSGPSGT |  |  | 4 |  |  |  | 1 |  | 4 |  |  |  |  |  |
| 60 | SAQSGTSGTSGTSAQSGTSGTSGTSGTSGTSGPSGTSGTSGTSGTSGTSGP |  |  | 1 |  |  |  |  |  |  |  |  |  |  |  |
| 61 | SARSGTSGTSGTSGPSGPSGT |  |  | 1 |  |  |  |  |  |  |  |  |  |  |  |
| 62 | SAQSGASAQSGASAQSGASAQSGASAQSGTSGPSGPSGPSGT |  |  |  |  |  |  | 2 |  |  |  |  |  |  |  |
| 63 | SAQSGASAQSGTSAQSGTSAQSGTSGTSGPSGT |  |  |  |  |  |  | 1 |  | 1 |  |  |  |  |  |
| 64 | SAQSGTSGTSGTSAQSGTSAQSGTSGTSGTSGTSGPSGPSGT |  |  |  |  |  |  | 1 |  |  |  |  |  |  |  |
| 65 | SAQSGTSGTSGPSGTSGPSGPSGT |  |  |  |  |  |  | 1 |  |  |  |  |  |  |  |
| 66 | SAQSGTSGTSGTSGTSGTSGTSGTSGTSGTSGTSGTSGPSGPSGPSGT |  |  |  |  |  |  | 1 |  |  |  |  |  |  |  |
| 67 | SAQSGTSGTSGTSGTSGPSGPSGTSGTSGTSGTSGTSGTSGPSGT |  |  |  |  |  |  | 1 |  |  |  |  |  |  |  |
| 68 | SAQSGTSGTSGPSGTSGPSGTSGPSGPSGT |  |  |  |  |  |  | 2 |  | 1 |  |  |  |  |  |
| 69 | SAQSGTSGTSGTSGTSGTSGTSGTSGTSGTSGTSGTSGTSGPSGPSGPSGT |  |  |  |  |  |  | 1 |  |  |  |  |  |  |  |
| 70 | SAQSGASAQSGTSAQSGTSAQSGTSGTSGTSGT | 5 |  |  |  |  |  | 1 |  |  |  |  |  |  |  |
| 71 | SAQSGASAQSGTSGTSGTSAQSGTSGTSGTSGTSGTSGPSGPSGPSGT |  |  |  |  |  |  | 1 |  |  |  |  |  |  |  |
| 72 | SAQSGTSGTSGPSGTSGPSGTSGPSGTSGPSGPSGT |  |  |  |  |  |  | 1 |  |  |  |  |  |  |  |
| 73 | SAQSGTSGTSAQSGTSGTSGTSGTSGTSGTSGTSGPSGT |  |  |  |  |  |  | 1 |  |  |  |  |  |  |  |
| 74 | SAQSGTSGTSAQSGTSGTSGTSGTSGTSGTSGPSGPSGPSGT |  |  |  |  |  |  | 1 |  |  |  |  |  |  |  |
| 75 | SAQSGTSGTSAQSGTSGTSGTSAQSGTSGTSGTSGPSGPSGT |  |  |  |  |  |  | 1 |  |  |  |  |  |  |  |
| 76 | SAQSGTSGTSGTSGTSGPSGPSGPSGPSGPSGPSGPSGT |  |  |  |  |  |  | 1 |  |  |  |  |  |  |  |
| 77 | SAQSGASAQSGTSAQSGTSAQSGTSAQSGTSGTSGTSGTSGPSGT |  |  |  |  |  |  | 1 |  |  |  |  |  |  |  |
| 78 | SAQSGTSGTSGPSGT |  |  |  |  |  |  | 1 | 1 | 2 |  |  |  | 5 |  |
| 79 | SAQSGASAQSGTSGPSGPSGPSGPSGPSGT |  |  |  |  |  |  | 1 |  |  |  |  |  |  |  |
| 80 | SAQSGTSGTSGPSGTSGPSGTSGPSGTSGTSGPSGPSGT |  |  |  |  |  |  | 1 |  |  |  |  |  |  |  |
| 81 | SAQSGTSGTSGTSGPSGTSGPSGTSGPSGTSGPSGTSGPSGTSGPSGPSGT |  |  |  |  |  |  | 1 |  |  |  |  |  |  |  |
| 82 | SAQSGTSGTSGTSAQSGTSAQSGTSAQSGTSGTSGTSGPSGPSGT |  |  |  |  |  |  | 1 |  |  |  |  |  |  |  |
| 83 | SAQSGTSGTSGTSGPSGTSGPSGTSGPSGTSGPSGPSGT |  |  |  |  |  |  | 1 |  |  |  |  |  |  |  |
| 84 | SAQSGTSGTSGPSGTSGPSGTSGPSGPSGTSGPSGPSGT |  |  |  |  |  |  |  |  |  |  | 5 |  |  |  |
| 85 | SAQSGTSGTSGTSAQSGTSAQSGTSGTSAQSGTSGTSGTSGTSGPSGPSGT |  |  |  |  |  |  |  |  |  |  | 4 |  |  |  |
| 86 | SAQSGTSGTSGTSGTSGTSGTSGTSGTSGTSGTSGTSGTSGTSGTSGPSGPSGT |  |  |  |  |  |  |  |  |  |  | 3 |  |  |  |
| 87 | SAQSGTSGTSGPSGTSGPSGTSGPSGTSGPSGTSGPSGT |  |  |  |  |  |  |  | 1 |  |  |  |  | 11 |  |
| 88 | SAQSGASAQSGASAQSGASGT |  |  |  |  |  |  |  | 1 |  |  |  |  |  |  |
| 89 | SAQSGASAQSGTSAQSGTSGTSGTSGPSGPSGT |  |  |  |  |  |  |  | 1 |  |  |  |  |  |  |
| 90 | SAQSGASAQSGASAQSGTSAQSGTSAQSGTSGTSGTSGTSGTSGPSGPSGPSGT |  |  |  |  |  |  | 1 |  |  |  |  |  |  |  |
| 91 | SAQSGTSGTSGTSGTSGTSGTSAQSGTSGTSGTSGTSGTSGPSGT |  |  |  |  |  |  | 1 |  | 3 |  |  |  |  |  |
| 92 | SAQSGTSGTSGTSGPSGPSGPSGPSGTSGT |  |  |  |  |  |  | 1 |  |  |  |  |  |  |  |
| 93 | SAQSGASAQSGASAQSGASAQSGASAQSGTSGPSGT |  |  |  |  |  |  | 1 |  | 1 |  |  |  |  |  |
| 94 | SAQSGPSGTSGPSGTSGPSGTSGPSGTSGPSGTSGPSGTSGPSGT |  |  |  |  |  |  | 1 |  |  |  |  |  |  |  |
| 95 | SAQSGASAQSGASAQSGTSAQSGTSAQSGTSGTSGTSGTSGPSGT |  |  |  |  |  |  | 1 |  |  |  |  |  |  |  |
| 96 | SAQSGTSGTSGTSAQSGTSGTSGTSAQSGTSGTSGPSGT |  |  |  |  |  |  | 1 |  |  |  |  |  |  |  |
| 97 | SAQSGASAQSGTSAQSGTSAQSGTSGTSGTSGTSGTSGTSGTSGT |  |  |  |  |  |  | 1 |  |  |  |  |  |  |  |
| 98 | SAQSGASAQSGTSAQSGTSAQSGTSGTSGPSGPSGT |  |  |  |  |  |  | 1 |  |  |  |  |  |  |  |
| 99 | SAQSGTSGTSGTSGTSGTSGTSAQSGTSGTSAQSGTSGTSGTSGPSGPSGT |  |  |  |  |  |  | 1 |  |  |  |  |  |  |  |
| 100 | SAQSGASAQSGASAQSGASAQSGASAQSGASAQSGASAQSGASAQSGTSGPSGT |  |  |  |  |  |  | 2 |  |  |  |  |  |  |  |
| 101 | SAQSGTSGTSGTSGTSAQSGTSGTSGTSGTSGTSGPSGT |  |  |  |  |  |  | 1 |  |  |  |  |  |  |  |
| 102 | SAQSGTSGTSGTSGTSGTSGTSGTSGPSGTSGTSGPSGPSGPSGT |  |  |  |  |  |  | 1 |  |  |  |  |  |  |  |
| 103 | SAQSGTSGTSGTSAQSGTSAQSGTSGPSGPSGT |  |  |  |  |  |  | 1 |  |  |  |  |  |  |  |
| 104 | SAQSGTSGTSGTSAQSGTSAQSGTSAQSGTSAQSGTSGPSGPSGPSGPSGT |  |  |  |  |  |  | 1 |  |  |  |  |  |  |  |
| 105 | SAQSGASAQSGTSAQSGTSAQSGTSAQSGTSGTSGPSGT |  |  |  |  |  |  | 1 |  |  |  |  |  |  |  |
| 106 | SAQSGTSGTSGTSGTSGTSGTSAQSGTSGTSGTSGTSGPSGT |  |  |  |  |  |  | 1 |  |  |  |  |  |  |  |
| 107 | SAQSGASAQSGTSAQSGTSAQSGTSGTSGTSGTSGTSGTSGTSG |  |  |  |  |  |  | 1 |  |  |  |  |  |  |  |
| 108 | SAQSGASAQSGASAQSGASAQSGASAQSGASAQSGASAQSGTSGPSGPSGPSGT |  |  |  |  |  |  | 1 |  |  |  |  |  |  |  |
| 109 | SAQSGTSGTSGTSGPSGT |  |  |  |  |  |  | 1 |  |  |  |  |  |  |  |
| 110 | SAQSGASAQSGTSGTSGTSGPSGPSGT |  |  |  |  |  |  | 1 |  |  |  |  |  |  |  |
| 111 | SAQSGAGAQSGASAQSGASAQSGASAQSGTSGPSGPSGT |  |  |  |  |  |  | 1 |  |  |  |  |  |  |  |
| 112 | SAQSGTSGTSGTSGTSGTSAQSGTSGTSGTSGTSGTSGTSGTSGTSGTSGPSGT |  |  |  |  |  |  | 1 |  |  |  |  |  |  |  |
| 113 | SAQSGASAQSGTSAQSGTSAQSGTSGTSGTSGPSGT |  |  |  |  |  |  | 1 |  |  |  |  |  |  |  |
| 114 | SAQSGTSGTSGTSGTSAQSGTSGTSGTSGPSGPSGPSGT |  |  |  |  |  |  | 1 |  |  |  |  |  |  |  |
| 115 | SAQSGTSGTSGTSAQSGTSGTSGTSGTSGTSGPSGT |  |  |  |  |  |  | 1 |  |  |  |  |  |  |  |
| 116 | SAQSGASAQSGTSAQSGTSAQSGTSGTSGTSGPSGPSGT |  |  |  |  |  |  | 1 |  |  |  |  |  |  |  |
| 117 | SAQSGTSGTSGTSGTSGTSGTSGTSGTSGTSGTSGTSGT |  |  |  |  |  |  | 1 |  |  |  |  |  |  |  |
| 118 | SAQSGTSGTSGTSGPSGPSGPSGPSGPSGP |  |  |  |  |  |  | 1 |  |  |  |  |  |  |  |
| 119 | SAQSGASAQSGTSAQSGTSGPSGTSGPSGTSGPSGTSGPSGPSGT |  |  |  |  |  |  | 1 |  |  |  |  |  |  |  |
| 120 | SAQSGTSGTSGTSGTSGTSGTSGTSGPSGPSGPSGT |  |  |  |  |  |  | 1 |  |  |  |  |  |  |  |
| 121 | SAQSGTSGTSGTSAQSGTSGTSGTSAQSGTSGTSGPSGPSGT |  |  |  |  |  |  | 1 |  |  |  |  |  |  |  |
| 122 | SAQSGTSGTSGTSGTSGTSGTSGTSGTSGTSAQSGTSGTSGT  SGTSGTSGTSGPSGT |  |  |  |  |  |  | 1 |  |  |  |  |  |  |  |
| 123 | SVQSGASAQSGASAQSGASAQSGTSGPSGPSGT |  |  |  |  |  |  | 1 |  |  |  |  |  |  |  |
| 124 | SAQSGTSGTSGTSGTSGTSGTSGTSGTSGTSGTSGTSGTSGPSGT |  |  |  |  |  |  |  |  | 1 |  |  |  |  |  |
| 125 | SAQSGTSGTSGTSGTSGTSGTSGTSGTSGTSGTSGTSGTSGPSGPSGT |  |  |  |  |  |  |  |  | 2 |  |  |  |  |  |
| 126 | SAQSGTSGTSGTSGTSGTSGTSGPSGT |  |  |  |  |  |  |  |  | 2 |  |  |  |  |  |
| 127 | SAQSGASAQSGTSGTSGTSGTSGPSGPSGT |  |  |  |  |  |  |  |  | 2 |  |  |  |  |  |
| 128 | SAQSGTSGTSGPSGTSGPSGTSGPSGTSGPSGPSGPSGT |  |  |  |  |  |  |  |  | 2 |  |  |  |  |  |
| 129 | SAQSGTSGTSGPSGTSGPSGT |  |  |  |  |  |  |  |  | 2 |  |  |  |  |  |
| 130 | SAQSGASAQSGASAQSGASAQSGASAQSGTSGPSGTSGPSGPSGT |  |  |  |  |  |  |  |  | 1 |  |  |  |  |  |
| 131 | SAQSGTSGTSGTSGTSGPSGTSGT |  |  |  |  |  |  |  |  | 2 |  |  |  |  |  |
| 132 | SAQSGTSGTSGPSGTSGTSGTSAQSGTSGTSAQSGTSGTSGTSGTSGTSGPSGT |  |  |  |  |  |  |  |  | 1 |  |  |  |  |  |
| 133 | SAQSGASAQSGTSAQSGTSAQSGTSGTSGTSGTSGTSGTSGTSGT  SGTSGTSGTSGPSGPSGT |  |  |  |  |  |  |  |  | 1 |  |  |  |  |  |
| 134 | SAQSGTSGPSGPSGPSGPSGPSGPSGPSGPSGT |  |  |  |  |  |  |  |  | 1 |  |  |  |  |  |
| 135 | SAQSGTSGTSGTSGPSGTSGPSGTSGTSGTSGTSGP |  |  |  |  |  |  |  |  | 1 |  |  |  |  |  |
| 136 | SAQSGASAQSGASAQSGTSGTSGTSGTSGPSGPSGT |  |  | 2 |  |  |  |  |  | 1 |  |  |  |  |  |
| 137 | SAQSAQSGTSGTSGTSGTSGTSGTSGTSAQSGTSAQSGTSGPSGPSGT |  |  |  |  |  |  |  |  | 1 |  |  |  |  |  |
| 138 | SAQSGASAQSGASAQSGASAQSGASAQSGASAQSGTSGPSGPSGPSGT |  |  |  |  |  |  |  |  | 1 |  |  |  |  |  |
| 139 | SAQSGTSGTSGTSGTSGPSGTSGPSGT |  |  |  |  |  |  |  |  | 1 |  |  |  |  |  |
| 140 | SAQSGTSGTSGTSGTSGTSGTSAQSGTSGTSAQSGTSGTSGPSGT |  |  |  |  |  |  |  |  | 1 |  |  |  |  |  |
| 141 | SAQSGTSGTSGPSGTSGTSGTSGTSAQSGTSGTSAQSGTSGTSGTSGPSGT |  |  |  |  |  |  |  |  | 1 |  |  |  |  |  |
| 142 | SAQSGTSGTSGPSGPSGPSGPSGT |  |  |  |  |  |  |  |  | 1 |  |  |  |  |  |
| 143 | SAQSGTSGTSGTSAQSGTSAQSGTSGTSGTSAQSGTSGTSGPSGT |  |  |  |  |  |  |  |  | 1 |  |  |  |  |  |
| 144 | SAQSGASAQSGTSAQSGTSGPSGPSGT |  |  |  |  |  |  |  |  | 1 |  |  |  |  |  |
| 145 | SAQSGASAQNGASAQSGASAQSGTSGPSGT |  |  |  |  |  |  |  |  | 1 |  |  |  |  |  |
| 146 | SAQSGTSGTSGTSGTSGTSGTSGTSGTSGPSGPSGPSGPSGT |  |  |  |  |  |  |  |  | 1 |  |  |  |  |  |
| 147 | SAQSGTSGTSGTSGTSGTSGTSGTSGTSGTSGTSGPSGPSGPSGT |  |  |  |  |  |  |  |  | 1 |  |  |  |  |  |
| 148 | SAQSGTSGTSGTSGTSGTSGTSGTSGTSGTSGTSGTSGTSGTSGTSAQSGTSGT  SAQSGTSGTSGTSGTSGTSGT |  |  |  |  |  |  |  |  | 1 |  |  |  |  |  |
| 149 | SAQSGTSGTSGTSGTSGTSGTSGTSGTSGTSAQSGTSGTSGTSAQSGT  SGTSGTSGTSGTSGPSGPSGT |  |  |  |  |  |  |  |  | 1 |  |  |  |  |  |
| 150 | SAQSGTSGTSGTSGSSGPSGT |  |  | 1 |  |  |  |  |  |  |  |  |  |  |  |
| 151 | SAQSGASAQSGASAQSGTSAQSGTSGTSGPSGPSGT |  |  | 1 |  |  |  |  |  |  |  |  |  |  |  |
| 152 | SAQSGTSGTSAQSGTSGTSGTSGTSGTSGTSGPSGPSGT |  |  | 1 |  |  |  |  |  |  |  |  |  |  |  |
|  | Total | 54 | 28 | 86 | 10 | 7 | 10 | 71 | 4 | 57 | 21 | 22 | 8 | 23 | 29 |
