# Supplementary material for: Changing pattern of the genetic diversities of Plasmodium falciparum merozoite surface protein-1 and merozoite surface protein-2 in Myanmar isolates
Source: Malar J. 2019 Jul 16;18:241. doi: 10.1186/s12936-019-2879-7 (PMC6636015; doi:10.1186/s12936-019-2879-7)
Supplement: Supplementary file 3 — Additional file 3: Table S3. Distribution of MAD20 alleles among global pfmsp-1. [file 12936_2019_2879_MOESM3_ESM.docx]

**Table S3. Distribution of MAD20 alleles among global pfmsp-1**

| No. | Alleles | Myanmar (04-06) | Myanmar (13-15) | India | Thailand | Vietnam | Philippines | Kenya | Tanzania | Ghana | PNG | Solomon Islands | Vanuatu | Brazil | Peru |
| --- | --- | --- | --- | --- | --- | --- | --- | --- | --- | --- | --- | --- | --- | --- | --- |
| 1 | Allele 9 (SGGSVASGG) |  | 1 |  |  |  |  |  |  |  |  |  |  |  |  |
| 2 | Allele 10 (SKGSVTSGG) |  | 1 |  |  |  |  |  |  |  |  |  |  |  |  |
| 3 | Allele 11 SKGSGGSVASVASGG) |  | 1 |  |  |  |  |  |  |  |  |  |  |  |  |
| 4 | Allele 12 (SGGSVTSGGSVTSGGSGGSVASGG) |  | 1 |  |  |  |  |  |  |  |  |  |  |  |  |
| 5 | Allele 13 (SKGSGGSVASGGSGGSGGSVASVASGG) |  | 1 |  |  |  |  |  |  |  |  |  |  |  |  |
| 6 | Allele 14 (SVTSGGSVTSGGSVTSVASVASVASVASGG) |  | 1 |  | 3 |  |  |  |  |  |  |  |  |  |  |
| 7 | Allele 15 (SGGSVTSGGSVTSGGSGGSVASVASVASGG) |  | 2 | 3 |  | 4 | 4 |  |  |  |  |  |  |  |  |
| 8 | Allele 16 (SGGSVTSGGSGGSVASVASGGSGGSVASGG) | 3 | 9 | 2 |  |  |  |  |  |  |  |  |  |  |  |
| 9 | Allele 17 (SGGSVTSGGSVTSGGSVTSVASVASVASVASGG) | 7 | 4 |  |  |  |  |  |  |  |  |  |  |  |  |
| 10 | Allele 18 (SGGSVTSGGSVTSGGSGGSVASVASVASVASGG) | 5 | 3 |  | 19 | 8 |  |  |  |  |  |  |  | 1 |  |
| 11 | Allele 19 (SKGSGGSVASGGSGGSGGSVASVASGGSVASVASGG) | 13 | 24 | 2 |  |  |  |  |  |  |  |  |  |  |  |
| 12 | Allele 20 (SKGSVTSGGSGGSGGSVASGGSVASGGSGGSVASVASGG) |  | 2 | 1 |  |  |  |  |  |  |  |  |  |  |  |
| 13 | Allele 21 (SKGSVTSGGSGGSGGSVASGGSGGSVASVASGGSVASGG) |  | 1 | 1 |  |  |  |  |  |  |  |  |  |  |  |
| 14 | Allele 22 (SKGSVASGGSVASGGSVASGGSGGSVASGGSGGSVASGG) |  | 1 |  |  |  |  |  |  | 1 |  |  |  |  |  |
| 15 | SGGSVTSGGSGGSGGSGGSVASGGSVASGGSGGSVASGGSVASGG |  |  |  |  |  |  |  |  |  |  |  |  | 10 | 2 |
| 16 | SGGSVTSGGSGGSGGSGGSGASGGSVASGGSGGSVASGGSVASGG |  |  |  |  |  |  |  |  |  |  |  |  | 3 |  |
| 17 | SGGSGTSGGSGGSGGSGGSGASGGSGASGGSGGSGASGGSVASGG |  |  |  |  |  |  |  |  |  |  |  |  | 1 |  |
| 18 | SKGSVASGGSGGSVASGGSVASGG |  |  |  |  |  |  |  |  |  |  |  |  | 1 |  |
| 19 | SKGSVTSGGSGGSVASGGSGGSVASGGSGGSVASGGSGGSVASGG |  |  |  |  |  |  |  |  | 2 |  |  |  |  |  |
| 20 | SKGSGGSVASGGSGGSVASGGSGGSVASGGSGGSVASGGSVASGGSVASGG |  |  |  |  |  |  |  |  | 2 |  |  |  |  |  |
| 21 | SKGSVASGGSVASGGSVASGGSVASGGSGGSVASGGSGGSVASGGSVASGG |  |  |  |  |  |  |  |  | 1 |  |  |  |  |  |
| 22 | SKGSVASGGSVASGGSVASGGSGGSGGSVASGGSVASGG |  |  |  |  |  |  |  |  | 1 |  |  |  |  |  |
| 23 | SGGSVTSGGSGGSGGSVASGGSVASGGSGGSVASGGSVASGG |  |  |  |  |  |  |  |  | 1 |  |  |  |  |  |
| 24 | SKGSVTSGGSGGSGGSGGSGG |  |  |  |  |  |  |  |  | 1 |  |  |  |  |  |
| 25 | SGGSVTSGGSGGSGGSGGSGGSVASGGSVASGGSGGSVASGGSVASGG |  |  |  |  |  |  |  |  |  |  |  |  |  | 24 |
| 26 | SVTSGGSGGSVASGGSGGSVASGGSGGSVASGG |  |  |  |  | 3 |  |  |  |  |  |  | 13 |  |  |
| 27 | SVASGGSVASGGSGGSVASGGSGGSVASGGSVASGG |  |  | 2 | 1 |  |  | 1 | 3 |  |  |  |  |  |  |
| 28 | SKGSGGSVASGGSGGSVASVASGGSVASVASGG |  |  |  |  | 1 | 2 |  |  |  |  |  |  |  |  |
| 29 | SVTSGGSGGSVASVASGGSGGSVASGG |  |  |  | 2 |  | 20 |  |  |  | 7 |  |  |  |  |
| 30 | SVTSGGSGGSGGSVASVASGGSGGSVASGG |  |  |  |  |  | 1 |  |  |  |  |  |  |  |  |
| 31 | SGGSVTSGGSGGSVASVASVASVASGG |  |  |  |  |  | 3 |  |  |  |  |  |  |  |  |
| 32 | SKGSVTSGGSGGSVASGGSGGSVASGGSGGSVASGGSVASGG |  |  | 3 |  | 7 | 1 | 1 |  |  |  |  |  |  |  |
| 33 | SVASGGSVASGGSVASGGSVASGGSVASGGSVASGGSVASGG |  |  |  |  |  |  |  |  |  |  | 2 |  |  |  |
| 34 | SVTSGGSGGSVASVASVASGGSGGSVASGG |  |  |  |  |  |  |  |  |  | 13 | 2 |  |  |  |
| 35 | GSSGSVTSGGSGGSVASGGSGGSVASGG |  |  |  |  |  |  |  |  |  | 2 | 16 |  |  |  |
| 36 | SVTSGGSGGSVASVASGGSVASGG |  |  |  |  |  |  |  |  |  | 2 | 1 |  |  |  |
| 37 | GSSGSVTSGGSGGSVASVASGGSGGSVASGG |  |  |  |  |  |  |  |  |  |  | 2 |  |  |  |
| 38 | SKGSVTSGGSGG |  |  |  |  |  |  | 1 |  |  |  |  |  |  |  |
| 39 | SKGSVTSGGSGGSVASGGSGGSVASGGSVASGGSVASGGSVASGG |  |  |  |  |  |  | 1 |  |  |  |  |  |  |  |
| 40 | SKGSVTSGGSGGSVASGGSVASGGSVASGGSVASGGSVASGG |  |  |  |  |  |  | 1 |  |  |  |  |  |  |  |
| 41 | SKGSVASGGSGGSVASGGSGGSVASGGSVASGGSVASGG |  |  |  |  |  |  | 1 |  |  |  |  |  |  |  |
| 42 | SKGSVASGGSGGSGGSVASGGSGGSVASGG |  |  |  |  |  |  | 1 |  |  |  |  |  |  |  |
| 43 | SKGSVTSGGSGGSVASGG |  |  |  |  |  |  | 1 |  |  |  |  |  |  |  |
| 44 | SKGSVASGGSGGSGGSVASGGSGGSVASGG |  |  |  |  |  |  |  |  |  |  |  |  |  |  |
| 45 | SKGSVTSGGSGGSVASGG |  |  |  |  |  |  |  |  |  |  |  |  |  |  |
| 46 | SKGSVTSGGSGGSVASGGSGGSVASGGSVASGG |  |  |  |  |  |  | 1 |  |  | 1 |  |  |  |  |
| 47 | SKGSVTSGGSGGSGG |  |  | 3 |  |  |  | 3 |  |  |  |  |  |  |  |
| 48 | SKGSVASGGSVASGGSVASGGSVASGGSVASGGSVASGG |  |  |  |  |  |  | 2 | 1 |  |  |  |  |  |  |
| 49 | SKGSVTSGGSGGSGGSVASGGSGGSVASGGSVASGG |  |  |  |  |  |  | 1 |  |  |  |  |  |  |  |
| 50 | SGGSVASGGSGGSVASGGSGGSVASGG |  |  |  |  |  |  | 1 |  |  |  |  |  |  |  |
| 51 | SKGSVTSGGSGGSGGSGG |  |  |  |  |  |  | 1 |  |  |  |  |  |  |  |
| 52 | SKGSVTSGGSGGSVASGGSGGSVASGGSVASGGSVASGG |  |  |  |  |  |  | 1 | 3 |  |  |  |  |  |  |
| 53 | SKGSVASGGSGGSVASGGSGGSVASGGSVASGG | 11 |  |  |  |  |  | 1 |  |  |  |  |  |  |  |
| 54 | SVTSGGSGGSVASGGSVASGG |  |  |  |  |  |  |  |  |  | 3 |  |  |  |  |
| 55 | SGGSVTSGGSGGSVASGGSGGSVASGGSGGSVASGGSVASGG |  |  |  |  |  |  |  | 4 |  | 10 |  |  |  |  |
| 56 | SGGSVTSGGSVTSGGSGGSGGSVASVASVASGG |  |  |  |  |  |  |  |  |  | 1 |  |  |  |  |
| 57 | SGGSVTSGGSGGSVASGGSGGSVASGGSGGSVASGG |  |  |  |  |  |  |  |  |  | 3 |  |  |  |  |
| 58 | SVTSGGSVASVASVASGGSGGSVASGG |  |  |  |  |  |  |  |  |  | 6 |  |  |  |  |
| 59 | SVTSGGSGGSVASGGSGGSVASGG |  |  |  |  |  |  |  |  |  | 1 |  |  |  |  |
| 60 | SKGSVASSGSVASGG |  |  |  | 2 |  |  |  |  |  |  |  |  |  |  |
| 61 | SGGSVTSGGSVTSGGSVTSVASVASVASVASVASGG | 18 |  |  | 3 |  |  |  |  |  |  |  |  |  |  |
| 62 | SVTSGG |  |  |  | 1 |  |  |  |  |  |  |  |  |  |  |
| 63 | SGGSVASGGSGGSVASGGSGGSVASGGSGGSVASGGSVASGG |  |  |  |  |  |  |  | 1 |  |  |  |  |  |  |
| 64 | SGGSVTSGGSGGSVASGGSGGSVASGGSVASGGSVASGGSVASGG |  |  |  |  |  |  |  | 1 |  |  |  |  |  |  |
| 65 | SKGSGGSVASGGSGGSVASGGSGGSVASGGSVASGGSVASGGSVASGG |  |  |  |  |  |  |  | 1 |  |  |  |  |  |  |
| 66 | SKGSVTSGGSGGSVASGGSGGSVASGGSGGSVASGGSVASGGSGGSVASGGSVASGG |  |  |  |  |  |  |  | 1 |  |  |  |  |  |  |
| 67 | SKGSVTSGGSGGSGGSVASGGSVASGGSVASGGSVASGGSVASGGSVASGG |  |  |  |  |  |  |  | 1 |  |  |  |  |  |  |
| 68 | SKGSVTSGGSGG |  |  |  |  |  |  |  | 1 |  |  |  |  |  |  |
| 69 | SKGSGGSVASGGSGGSVASGGSGGSVASGGSVASGGSVASGG |  |  |  |  |  |  |  | 1 |  |  |  |  |  |  |
| 70 | SGGSVTSGGSGGSVASGGSSGSVASGGSGGSVASGGSVASGG |  |  |  |  |  |  |  | 1 |  |  |  |  |  |  |
| 71 | SGGSVTSGGSGTSGGSGGSVASVASVASGG |  |  |  |  | 10 |  |  |  |  |  |  |  |  |  |
| 72 | SKGSVASGGSGGSVASVASGGSVASGG |  |  |  |  | 1 |  |  |  |  |  |  |  |  |  |
| 73 | SKGSVTSGGSGGSVASGGSGGSVASGGSGGSVASGG |  |  | 3 |  | 5 |  |  |  |  |  |  |  |  |  |
| 74 | SKGSVASGGSGGSVASGGSGGSVASGGSVASGASVASGG |  |  |  |  | 2 |  |  |  |  |  |  |  |  |  |
| 75 | SKGSVTSGGSGGSVASGGSGASVASGGSGGSVASGGSVASGG |  |  |  |  | 1 |  |  |  |  |  |  |  |  |  |
| 76 | SVTSGGSGGSVASVASGGSGG |  |  |  |  | 1 |  |  |  |  |  |  |  |  |  |
| 77 | SGGSVTSGGSGGSVASVASGG | 6 |  | 3 |  |  |  |  |  |  |  |  |  |  |  |
| 78 | SGGSVTSGGSVTSGGSVASGG |  |  | 1 |  |  |  |  |  |  |  |  |  |  |  |
| 79 | SGGSVTSGGSGGSVASVASVASGG |  |  | 3 |  |  |  |  |  |  |  |  |  |  |  |
| 80 | SGGSVTSGGSVASVASGGSGGSVASGG |  |  | 1 |  |  |  |  |  |  |  |  |  |  |  |
| 81 | SKGSGGSVASVASGGSVASVASGGSGGSVASGG |  |  | 1 |  |  |  |  |  |  |  |  |  |  |  |
| 82 | SGGSVTSGGSGGSGGSVASVASGGSGGSVASGG |  |  | 1 |  |  |  |  |  |  |  |  |  |  |  |
| 83 | SKGSGGSVASGGSGGSGGSGGSVASVASGGSVASVASGG |  |  | 1 |  |  |  |  |  |  |  |  |  |  |  |
| 84 | SVASGGSVASGGSVASGGSGGSVASGGSGGSVASGGSVASGG |  |  | 1 |  |  |  |  |  |  |  |  |  |  |  |
| 85 | SKGSVTSGGSGGSVASGGSVASGGSVASGGSGGSVASVASGG |  |  | 1 |  |  |  |  |  |  |  |  |  |  |  |
| 86 | SKGSVTSGGSGGSGGSVASGGSVASGGSGGSVASVASGGSVASGG |  |  | 2 |  |  |  |  |  |  |  |  |  |  |  |
| 87 | SKGSVTSGGSGGSGGSGGSVASGGSVASGGSGGSVASVASGGSVASGG |  |  | 2 |  |  |  |  |  |  |  |  |  |  |  |
| 88 | SGGSVTSGGSGGSGGSGGSVASGG |  |  | 1 |  |  |  |  |  |  |  |  |  |  |  |
| 89 | SKGSVASGG |  |  | 1 |  |  |  |  |  |  |  |  |  |  |  |
| 90 | SKGSVGSGGSGGSVGSGGSGGSVASGGSGGSVASGG |  |  | 1 |  |  |  |  |  |  |  |  |  |  |  |
| 91 | SKGSVTSGGSGGSVASGGSGGSVASVASGGSVASGG |  |  | 1 |  |  |  |  |  |  |  |  |  |  |  |
| 92 | SVASGGSVASGGSGGSVASGGSGGSGGSVASGGSVASGG |  |  | 1 |  |  |  |  |  |  |  |  |  |  |  |
| 93 | SKGSGGSGGSGGSVASGGSGGSVASGGSGGSVASGG |  |  | 1 |  |  |  |  |  |  |  |  |  |  |  |
| 94 | SGGSVTSGGSGGSVASGG |  |  | 1 |  |  |  |  |  |  |  |  |  |  |  |
| 95 | SKGSGGSVASVTSGGSGGSVASVASGGSGGSVASGG | 7 |  |  |  |  |  |  |  |  |  |  |  |  |  |
| 96 | SKGSGGSGGSVASGGSGGSVASVASGGSVASGGSGGSGGSVASVASGGSVASVASGG | 4 |  |  |  |  |  |  |  |  |  |  |  |  |  |
| 97 | SKGSVTSGGSGGSVASGGSGGSVASGGSGGSVASGGSGGSVASGG |  |  |  |  |  |  |  |  | 1 |  |  |  |  |  |
|  | Total | 74 | 52 | 44 | 31 | 43 | 31 | 19 | 19 | 9 | 49 | 23 | 13 | 16 | 26 |
