# Supplementary material for: Changing pattern of the genetic diversities of Plasmodium falciparum merozoite surface protein-1 and merozoite surface protein-2 in Myanmar isolates
Source: Malar J. 2019 Jul 16;18:241. doi: 10.1186/s12936-019-2879-7 (PMC6636015; doi:10.1186/s12936-019-2879-7)
Supplement: Supplementary file 4 — Additional file 4: Fig. S1. Polymorphisms and distributions of RO33 alleles among global pfmsp-1. (a) Sequence alignment of RO33 allelic types among global pfmsp-1. The eighteen RO33 alleles were identified in global pfmsp-1. The dots represent residues identical to the reference sequence of RO33 (AB276005). Amino acid changes were marked with reds. (b) Frequency of RO33 alleles among global pfmsp-1. PNG, Papua New Guinea. [file 12936_2019_2879_MOESM4_ESM.pptx]

## Slide 1
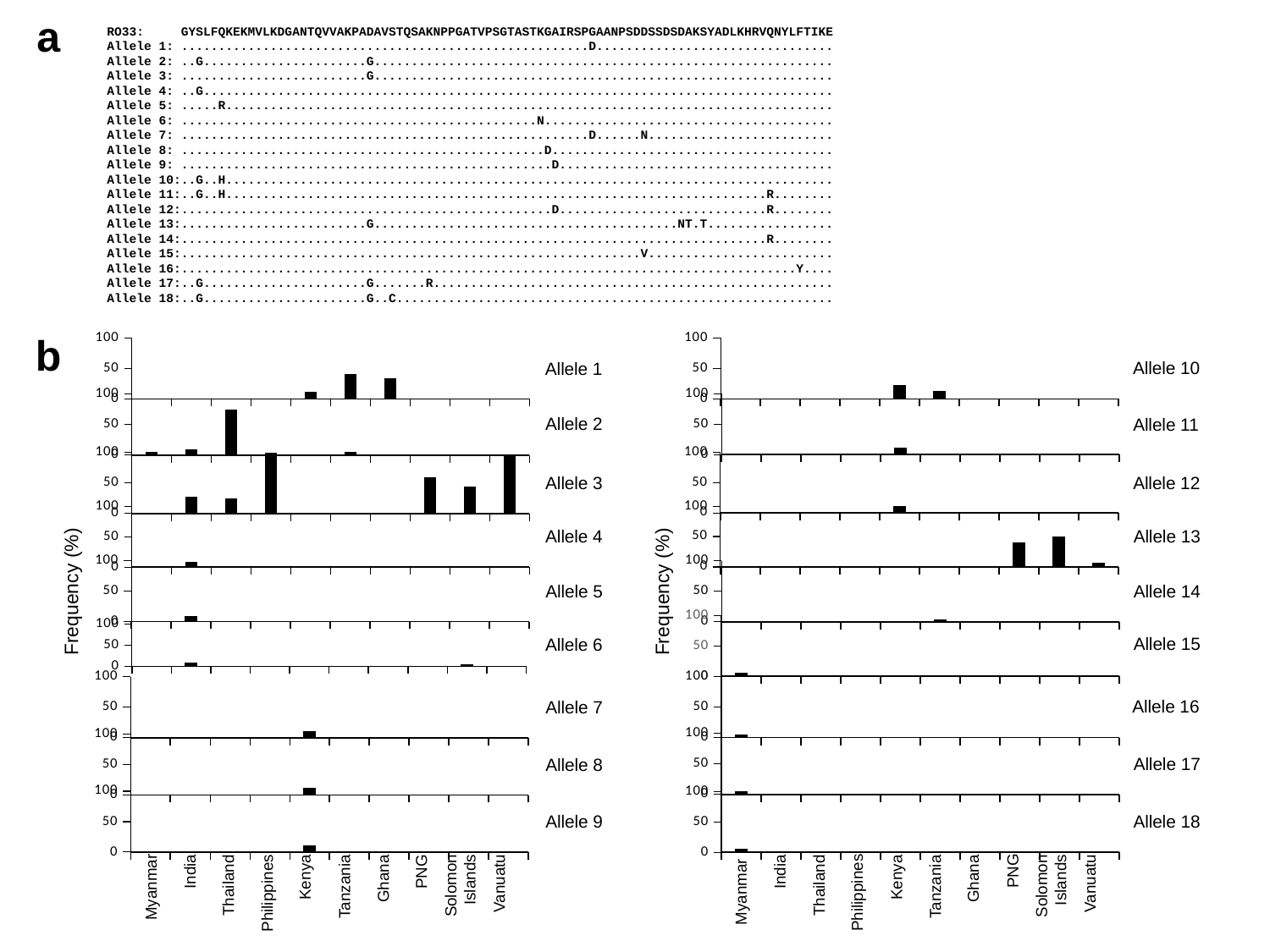

a
RO33: GYSLFQKEKMVLKDGANTQVVAKPADAVSTQSAKNPPGATVPSGTASTKGAIRSPGAANPSDDSSDSDAKSYADLKHRVQNYLFTIKE
Allele 1: .......................................................D................................
Allele 2: ..G......................G..............................................................
Allele 3: .........................G..............................................................
Allele 4: ..G.....................................................................................
Allele 5: .....R..................................................................................
Allele 6: ................................................N.......................................
Allele 7: .......................................................D......N.........................
Allele 8: .................................................D......................................
Allele 9: ..................................................D.....................................
Allele 10:..G..H..................................................................................
Allele 11:..G..H.........................................................................R........
Allele 12:..................................................D............................R........
Allele 13:.........................G.........................................NT.T.................
Allele 14:...............................................................................R........
Allele 15:..............................................................V.........................
Allele 16:...................................................................................Y....
Allele 17:..G......................G.......R......................................................
Allele 18:..G......................G..C...........................................................
b
### Chart
| Category | Allele 1 |
|---|---|
| Myanmar (2013-2016) | 0.0 |
| India | 0.0 |
| Thailand | 0.0 |
| Phillipines | None |
| Kenya | 11.111111111111102 |
| Tanzania | 40.909090909090914 |
| Ghana | 33.33333333333333 |
| PNG | 0.0 |
| Solomon Islands | 0.0 |
| Vanuatu | 0.0 |
### Chart
| Category | Allele 10 |
|---|---|Allele 10
Allele 1
### Chart
| Category | Allele 7 |
|---|---|
### Chart
| Category | Allele 2 |
|---|---|
| Myanmar (2013-2016) | 5.263157894736842 |
| India | 9.090909090909097 |
| Thailand | 75.0 |
| Phillipines | None |
| Kenya | 0.0 |
| Tanzania | 4.545454545454546 |
| Ghana | 0.0 |
| PNG | 0.0 |
| Solomon Islands | 0.0 |
| Vanuatu | 0.0 |Allele 2
Allele 11
### Chart
| Category | Allele 7 |
|---|---|
### Chart
| Category | Allele 3 |
|---|---|
| Myanmar (2013-2016) | 0.0 |
| India | 27.272727272727238 |
| Thailand | 25.0 |
| Phillipines | 100.0 |
| Kenya | 0.0 |
| Tanzania | 0.0 |
| Ghana | 0.0 |
| PNG | 60.0 |
| Solomon Islands | 44.4444444444444 |
| Vanuatu | 93.75 |Allele 3
Allele 12
### Chart
| Category | Allele 13 |
|---|---|
### Chart
| Category | Allele 4 |
|---|---|
| Myanmar (2013-2016) | 0.0 |
| India | 9.090909090909097 |
| Thailand | 0.0 |
| Phillipines | None |
| Kenya | 0.0 |
| Tanzania | 0.0 |
| Ghana | 0.0 |
| PNG | 0.0 |
| Solomon Islands | 0.0 |
| Vanuatu | 0.0 |Allele 13
Allele 4
### Chart
| Category | Allele 5 |
|---|---|
### Chart
| Category | Allele 14 |
|---|---|Frequency (%)
Frequency (%)
Allele 14
Allele 5
### Chart
| Category | Allele 15 |
|---|---|
### Chart
| Category | Allele 6 |
|---|---|Allele 15
Allele 6
### Chart
| Category | Allele 15 |
|---|---|
### Chart
| Category | Allele 7 |
|---|---|Allele 16
Allele 7
### Chart
| Category | Allele 15 |
|---|---|
### Chart
| Category | Allele 7 |
|---|---|Allele 17
Allele 8
### Chart
| Category | Allele 7 |
|---|---|
### Chart
| Category | Allele 15 |
|---|---|Allele 18
Allele 9
India
Solomon Islands
Solomon Islands
India
PNG
Ghana
PNG
Kenya
Kenya
Ghana
Vanuatu
Thailand
Vanuatu
Myanmar
Tanzania
Tanzania
Thailand
Myanmar
Philippines
Philippines
